# Circadian and circatidal clocks control the mechanism of semilunar foraging behaviour

**DOI:** 10.1038/s41598-017-03245-3

**Published:** 2017-06-19

**Authors:** James F. Cheeseman, Rachel M. Fewster, Michael M. Walker

**Affiliations:** 1 Department of Anaesthesiology, School of Medicine, The University of Auckland, Private Bag 92019, Auckland, 1142 New Zealand; 2 Department of Biological Sciences, The University of Auckland, Private Bag 92019, Auckland, 1142 New Zealand; 3 Department of Statistics, The University of Auckland, Private Bag 92019, Auckland, 1142 New Zealand

## Abstract

How animals precisely time behaviour over the lunar cycle is a decades-old mystery. Experiments on diverse species show this behaviour to be endogenous and under clock control but the mechanism has remained elusive. We present new experimental and analytical techniques to test the hypotheses for the semilunar clock and show that the rhythm of foraging behaviour in the intertidal isopod, *Scyphax ornatus*, can be precisely shifted by manipulating the lengths of the light/dark and tidal cycles. Using light T-cycles (T_cd_) the resultant semilunar beat period undergoes shifts from 14.79 days to 6.47 days under T = 23 hours (h), or to 23.29 days under T = 24.3 h. In tidal T-cycles (T_t_) of natural length T_t_ = 12.42 h, the semilunar rhythm is shifted to 24.5 days under T_t_ = 12.25 h and to 9.7 days under T_t_ = 12.65 h. The implications of this finding go beyond our model species and illustrate that longer period rhythms can be generated by shorter period clocks. Our novel analysis, in which periodic spline models are embedded within randomization tests, creates a new methodology for assessing long-period rhythms in chronobiology. Applications are far-reaching and extend to other species and rhythms, potentially including the human-ovarian cycle.

## Introduction

Persistent lunar (circa 29.5 day) and semilunar (circa 14.7 day) rhythms have been observed and described in plants, algae and animals for decades^1–3^. Laboratory experiments on diverse species have shown these behaviours to be endogenous^4–7^, under clock control, and able to be entrained^8^, but the mechanisms, physical location and molecular nature of the controlling clocks remain almost totally unknown^9^. This is in stark contrast to the study of the daily circadian clock which is well characterized in terms of behaviour, physiology and molecular clock gene machinery.

Field studies have described the accurate timing of lunar reproduction in marine organisms such as the Palolo worm (*Eunice viridis*)^5–7^, and laboratory studies of reproduction in the marine insects *Clunio* and *Pontomyia* have shown that the endogenous lunar clock is resistant to manipulations in period because it is temperature compensated^10, 11^. In *Clunio*, experimental studies have identified functional and pigment changes in the larval ocelli^12^ together with a photoreceptor pigment (ciliary opsin 2) and a known insect circadian clock gene *Cryptochrome1* that are now thought to be associated with the lunar rhythm^13^. Most recently the characterization of the *Clunio* genome has identified orthologues for the circadian clock genes but these do not yet explain the lunar mechanism^14^. For the semilunar clock, by contrast, three mechanisms have been proposed: (1) an endogenous clock analogous to the circadian clock but generated by an oscillation with an innate period of 15 days; (2) the beats hypothesis in which the combined outputs of a circadian clock and circatidal clock interact constructively and destructively to induce the longer period (circa 15 day) semilunar rhythms^4^; and (3) the frequency demultiplication hypothesis^15, 16^ in which changes in circadian period lead to proportional changes in the semilunar period.

One complication that arises in distinguishing between the three proposed mechanisms for the semilunar clock is the current lack of understanding of the molecular basis of the clocks controlling tidal (12.4 h) behaviour. Arguably the critical contemporary question is the neurobiological and molecular-genetic identity of these non-circadian clocks. Progress in this direction has included using circadian clock gene knockouts which disrupt circadian behaviour but not tidal behaviour and suggest the existence of an independent 12.4 hour oscillator^17–19^, and identifying potential metabolic markers of the tidal clock^20^. However, notwithstanding these tantalizing results, no candidate genes for the tidal clock have thus far been forthcoming. Furthermore, even the most recent molecular techniques, which inhibit the Casein kinase 1δ/ε enzyme to disrupt the circadian clock while leaving the circatidal and circalunar timing systems in place^7, 19^, would not naturally resolve the semilunar clock mechanism, because abolition of the circadian clock would remove the semilunar rhythm under at least two of the proposed semilunar mechanisms. In the absence of available molecular approaches for determining the mechanism of the semilunar clock, here we propose a behavioural approach. This approach has the additional advantage that it is directly relevant to applications such as aquaculture, in which it is the behavioural outcomes of the semilunar rhythm that are of primary interest.

**Figure 1:**
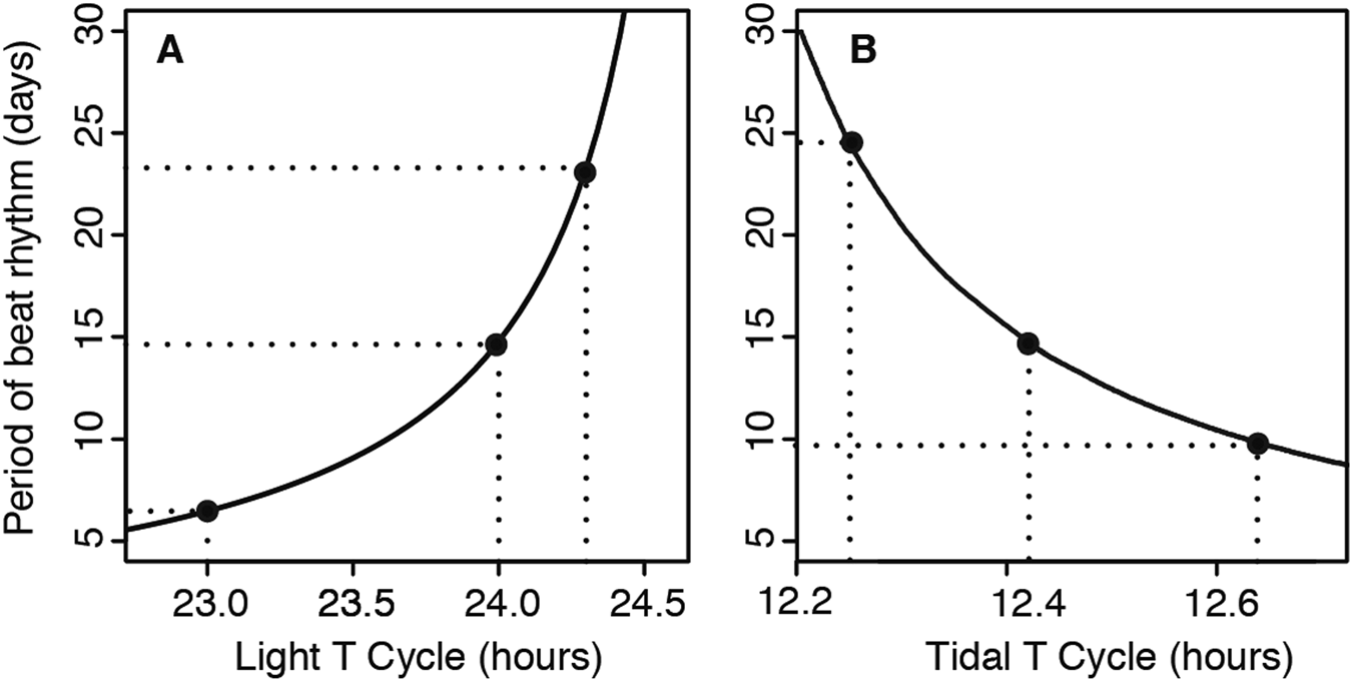
Predictions of the length of the semilunar rhythm under the beats hypothesis. The curves predict the period of the semilunar rhythm. In (**A**) the tidal cycle is kept constant at 12.42 hours while the Light T-cycle is varied. In (**B**) the 24 hour light cycle is kept constant while the Tidal T-cycle is varied. The circles represent the predicted semilunar periods for T-cycle experiments 1 to 6.

In contrast to previous studies, which focus on reproductive timing, commercial^21, 22^ and recreational fish catches suggest it should be possible to demonstrate a semilunar pattern in foraging activity. Semilunar modulation of daily activity has been demonstrated experimentally in several arthropods^4, 5, 23, 24^, and experimental study of foraging activity has the advantage that activity of individual animals can be recorded over multiple cycles of the semilunar rhythm, which is not possible when studying infrequent reproductive events. We therefore sought to use foraging behaviour to distinguish among the three hypotheses for the mechanism of the semilunar rhythm in a series of experiments using artificially long and short circadian and circatidal T-cycles (see Fig. 1).

We manipulated day length while keeping tide length constant, and *vice versa*, and examined the foraging behaviour of the New Zealand isopod, *Scyphax ornatus*, a species previously shown to exhibit persistent endogenous circadian and semilunar behaviour^5^. In their typical natural behaviour, *Scyphax* emerge from their burrows above the high tide line on exposed sandy beaches shortly after nightfall, move quickly to the water’s edge to feed vigorously on detritus^5^ that is washed up by the incoming tide, then return to the supra-littoral zone and burrow 20–30 cm under the sand at or before dawn, where they may remain for several days after feeding. Foraging time is determined by the duration of incoming tide after dark rather than by absolute tide height, and nights in which maximum foraging activity occurs need not be in phase with spring or neap tides. We tested the prediction from the beats hypothesis that the length of the semilunar behavioural rhythm will vary according to the interaction of the tidal and circadian cycles. This can be achieved by the equation^4^:

1 $${{\rm{T}}}_{{\rm{lunar}}}=\frac{{{\rm{T}}}_{{\rm{cd}}}\times {{\rm{2T}}}_{{\rm{t}}}}{|{{\rm{T}}}_{{\rm{cd}}}-{{\rm{2T}}}_{{\rm{t}}}|}$$

where T_cd_ and T_t_ are the period lengths of the manipulated circadian and circatidal clocks respectively, and where we report T_lunar_ in days of 24 hours’ duration for consistency. The semilunar period is ${{\rm{T}}}_{{\rm{lunar}}}/2$. By contrast, the hypothesis of an independent semilunar clock predicts no change in the period of foraging activity exhibited by *Scyphax* under experimental manipulation of T_cd_ and T_t_, and the frequency demultiplication hypothesis predicts a change in the period of foraging activity that is directly proportional to the changes induced in the circadian clock. We introduce a novel statistical methodology to assess these hypotheses, which we term periodic-spline randomization testing. This technique establishes a new way of studying long-period behavioural rhythms, which are typically undetected by traditional time-series analyses due to a lack of power when experiments can only encompass a small number of long-period cycles.

## Results

**Figure 2:**
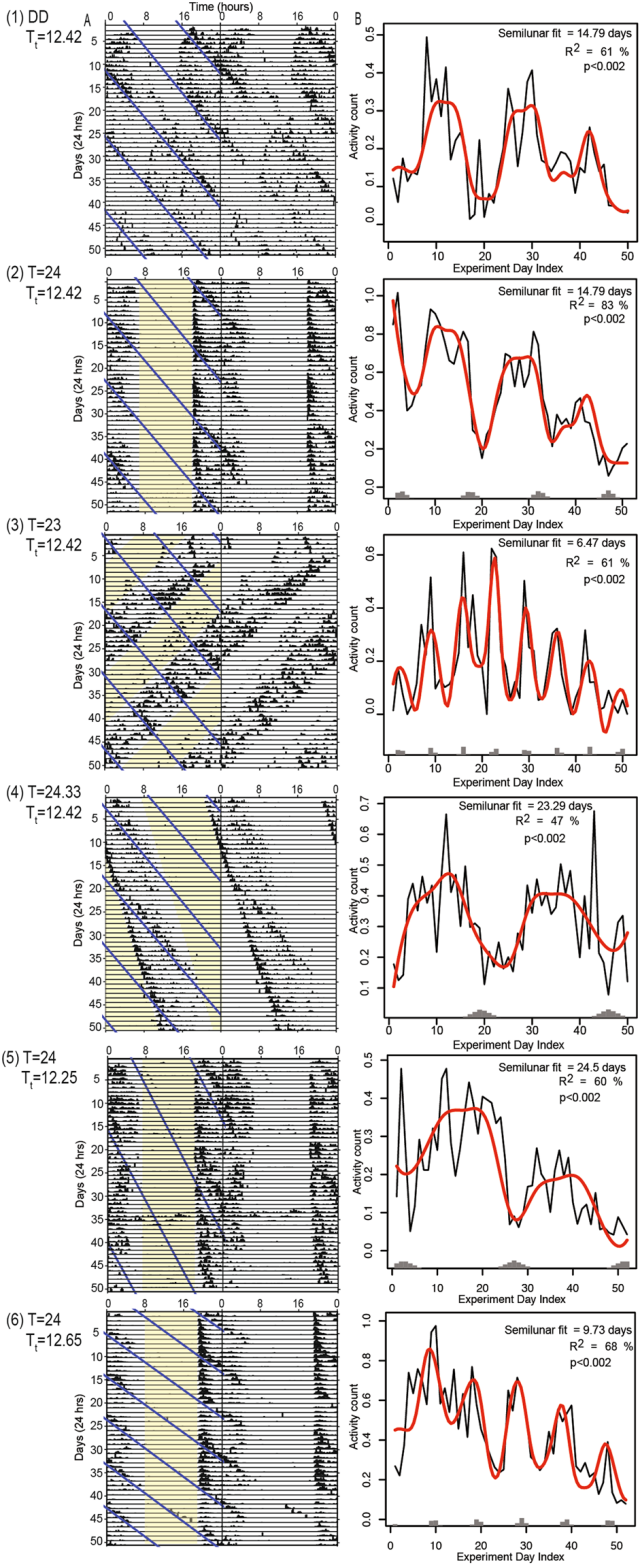
Behaviour of *Scyphax ornatus* in six test experiments (rows 1–6). (1) Constant darkness, n = 20; (2) T = 24 hours, n = 25; (3) T = 23 hours, n = 20; (4) T = 24.3 hours, n = 18; (5) Tidal T = 12.25 hours, n = 19; (6) Tidal T = 12.65 hours, n = 20. Column (**A**) shows double plotted activity plots (actograms). The actograms represent the combined total of the normalized individuals (see Supplementary Information S2 for all individual actograms). Black bars indicate activity. The lights and tide signals are shown in yellow and blue, respectively, on the left-hand panel only. Column (**B**) indicates the model fit with the beats-predicted period. The black line indicates the observed amount of *Scyphax* activity in the analysis window per night. The red line shows the model fit. Grey bars represent nights when the tidal signal coincided with the analysis window; heights of bars indicate the duration of coincidence. (See also S1 for periodogram analysis of the shorter period circatidal and circadian rhythms in the data).

In constant darkness (DD; Experiment 1), the circadian, circatidal, and semilunar behaviour persisted with a mean free running circadian peak at 23.83 h, close to the predicted 24 h, and with circatidal peaks at 12.42 h and 24.83 h (Chi-squared periodogram analysis of combined individuals S1, *n* = 20). The fit to the hypothesized semilunar period of 14.79 days was well-supported (*R* ^2^ 61%) and significantly better than chance (Randomization analysis: p < 0.002; Table 1; Fig. 2, panel 1B; S3). Actograms are shown in Fig. 2, panel 1A (summary) and S2 (all animals).

**Table 1:** The summary of experiments showing the light and tidal T-cycles, the predicted beat period and the outcome of each experiment.

Experiment	Light T-cycle (hours)	Tidal T-cycle (hours)	Predicted Period (days)	Best model fit (days)	R squared: Predicted	R squared: Best	*P* value
1	DD	12.42	14.79	16.3	61%	68%	<0.002
2	24 (LD12:12)	12.42	14.79	14.5	83%	84%	<0.002
3	23 (LD11.5:11.5)	12.42	6.47	6.5	61%	62%	<0.002
4	24.3 (LD12.15:12.15)	12.42	23.29	23.8	47%	48%	<0.002
5	24 (LD12:12)	12.25	24.50	23.0	60%	61%	<0.002
6	24 (LD12:12)	12.65	9.73	10.3	68%	72%	<0.002

In Experiment 2 (LD 12:12), animals readily entrained to a normal 24 h light dark cycle and the predicted semilunar modulation of their nocturnal activity on a 14.79-day basis was very well supported (*R* ^2^ 83%; p < 0.002; Fig. 2, panel 2B; S3). *Scyphax* often anticipated the tidal signal with an increase in activity in advance of the tidal onset (Fig. 2, panel 2A).

Experiments 3 and 4 exposed animals to artificially short (23 h) and long (24.3 h) light T-cycles that were chosen specifically to be within the circadian range of entrainment while predicting semilunar periods substantially shorter and longer (circa 6.47 and 23.29 days, respectively) than the circa 15-day norm. Animals showed stable entrainment to both light T-cycles with the semilunar behavioural rhythm being shortened to circa 6.47 days under T = 23 h (*R* ^*2*^ 61%, p < 0.002) and lengthened to circa 23.29 days under T = 24.3 h (*R* ^*2*^ 47%, p < 0.002) (Fig. 2; S3). In each case, the predicted semilunar period under the beats hypothesis was in the region best supported by the *R* ^*2*^ statistic (S3). Under T = 24.3 h we obtained a single higher *R* ^*2*^ value for a period of much lower duration (around six days) (S4); we regard this as a statistical anomaly as it does not support any of the hypotheses for semilunar periodicity.

In the Tidal T-cycle experiments there was a power-cut during the dark phase on day 20 (Experiment 5) and day 22 (Experiment 6) which resulted in several hours of missing data and a tidal stimulus not being given to the animals during those cycles. Data for these missing hours were imputed before conducting randomizations, using the mean of each animals’ activity during the equivalent hours in the two adjacent days. For the T_t_ = 12.25 experiment, the semilunar rhythm was extended as predicted. The best-fit model had a period of 23 days rather than the predicted 24.5 days, but by only the smallest of differences in *R* ^*2*^ (61% versus 60%: Table 1; S4), and the fit was substantially better supported under the beats hypothesis (*R* ^*2*^ 60%) than under the hypothesis of a 14.79-day endogenous clock (*R* ^*2*^ 41%). Similarly, under T_t_ = 12.65 the period with the highest *R* ^*2*^ fit was 10.3 days (*R* ^*2*^ 72%), fractionally higher than the predicted 9.73 day period (*R* ^*2*^ 68%: Table 1; S3 and S4).

Across all experiments, the peaks of the activity traces did not typically occur at times when the 120-minute analysis windows coincided with the 120-minute tidal stimulus (Fig. 2, Column B grey bars). Only in Experiments 3 and 6 (T = 23; T_t_ = 12.65) was the window close. This suggests that the results observed across the experiments are not due to masking of the underlying clock.

In each of the six experiments we tested whether the appearance of periodicity at the period predicted by the beats hypothesis could be artificially created by non-rhythmic effects common to all animals by randomizing all animals collectively for each simulation. Each of the experiments yielded strong evidence that the periodic signal was not created by external influences common to all animals (Experiments 1–6 p < 0.002; Scheme 2, S3). Finally we tested to what extent animals were in phase with one another by randomizing the start day and thereafter preserving the day order. In Experiment 1 (DD) and Experiment 4 (T24.3), the p-value did not reach significance (p = 0.16 and p = 0.26 respectively) suggesting that artificial re-phasing of individual animals could improve the periodic fit in these experiments. For Experiments 2 (T24) and 3 (T23) the highly significant results gave evidence of a common phasing among animals (p < 0.002; Scheme 3, S3), while the Tidal T-cycle Experiments 5 (T_t_ = 12.25) and 6 (T_t_ = 12.65) each gave moderately significant evidence of common phasing (p = 0.05 and p = 0.01; Scheme 3, S3).

## Discussion

Our experiments strongly support the beats hypothesis for the circa-semilunar rhythm in *Scyphax*. Using both circadian and circatidal T-cycles we were able to shift the period of foraging activity extremely close to the predicted values. To our knowledge, using both light and tidal T-cycles has not been tested before in the same animal. Importantly the T-cycles that we used in all the experiments were never more than one hour different from the natural period (Tau) and therefore within the range of entrainment of the animal. This also meant that the behavioural periods predicted by the frequency demultiplication hypothesis would be indistinguishable from 14.7 days in every instance. Our evidence did not support a steady period of around 14.7 days. Furthermore our experiments do not rely on the mechanism of the tidal clock, so we do not have to make assumptions about whether the tidal clock comprises one 12.42 hour or two 24.84 hour oscillators in order to interpret our results.

In this work we have also proposed a new method of statistical analysis that is robust to the relatively small number of semilunar cycles available in foraging experiments lasting two months, where animals must remain unfed to promote foraging incentive. While generally accepted in many scientific fields, randomization techniques have not previously been used in chronobiological studies. Our work demonstrates the power of these techniques for tackling complex long-period rhythms which are not amenable to traditional time series analyses.

The beats model permits highly accurate predictions of the semilunar modulation of the foraging activity of *Scyphax*, whereas previous studies using T-cycles have produced ambiguous results. It was previously shown that the semilunar release of gametes in the brown alga, *Dictyota*, could be shifted from 15 days to either longer or shorter periods^25^, but not to the extent predicted by the beats model. When *Clunio* were exposed to artificial light dark (LD) cycles of 10:10 and 15:15, the lunar pattern was no longer clear^25^, possibly because the range of circadian entrainment had been exceeded^26^, or because lunar peaks were split owing to individual animals within the experimental population responding differently to the *zeitgebers* (time givers)^26^. The behaviour of *Scyphax* in the laboratory, by contrast, is consistent with its observed behaviour on its home beach^27^. *Scyphax* depends on an accurate circadian clock for it to be able to emerge at the appropriate time after several days in its burrow, where it is completely isolated from normal circadian time-giving cues. On emergence from its burrow early in the dark period, *Scyphax* subsequently forages for extended periods in association with the circatidal signal present at the time. This pattern of behaviour generates active and quiescent periods of varying lengths during the dark period and suggests a model in which the circadian clock initiates emergence but subsequent activity then depends on the output levels of the circatidal signals.

We found no evidence that putative external events outside our control caused the semilunar temporal patterns (Randomization Scheme 2; Experiments 1 to 6). When we tested for semilunar phase cohesion amongst animals, we found strong evidence that the animals were acting in phase in the T24 and T23 experiments (Experiments 2 and 3), and moderate evidence in the Tidal T_t_ = 12.25 and T_t_ = 12.65 experiments (Experiments 5 and 6). The lack of evidence of phase cohesion in Experiments 1 and 4 can be readily explained. In the absence of light cycles (DD, Experiment 1) the animals, while still free running with a circadian period close to 24 h, would over the course of the 50 day experiment drift out of phase with one another. Similarly it is possible that in the longer T-cycle (T24.3, Experiment 4) the extended dark period would allow for greater flexibility in the phase of individuals.

Our experimental setup in the laboratory is a simplification of what the animals contend with in the wild, and yet we saw a range of behaviours among individuals (S2). Foraging behaviour was never rewarded with food but individuals did not always forage every time there was an opportunity to do so and sometimes stayed buried for days at a time. This suggests that there may be other factors operating to increase residual variation in the behaviour observed. The Polynesian fishing calendars certainly suggest greater complexity in foraging behaviour than we demonstrated in the circa-semilunar rhythm of *Scyphax* in the laboratory, but the basis for such complexity is not yet known. Certainly the Polynesian fishing calendars, which focus on predicting capture of fish and other species over the lunar and annual cycles, successfully predict the mass spawning events such as that of the Palolo worm which have drawn the bulk of scientific analysis to date.

Our experiments demonstrate that the semilunar timing mechanism in *Scyphax* can be more plausibly explained by the combination of interacting circadian and circatidal clocks proposed in the beats hypothesis than by a separate independent 15-day oscillator or by the frequency demultiplication hypothesis. Our study is purely behavioural, but future work will no doubt uncover the molecular basis of the circatidal rhythm and its interaction with the circadian. In the interim, studies such as ours help to focus the search for molecular clock mechanisms by eliciting the oscillator periods that are necessarily involved in generating behavioural outcomes.

We have shown that *Scyphax* can use robust and repetitive tidal and circadian information to schedule semilunar modulation of its daily activities. We suggest that the beats mechanism could also explain the timing of highly synchronized spawning in species such as the Palolo worm and California grunion (*Leuresthes tenuis*). Furthermore, given that animals such as the mangrove cricket (*Apteronemobius asahinai*)^28, 29^ show endogenous circatidal rhythms, it is reasonable to hypothesize that a similar beats mechanism may explain endogenous lunar rhythms in other terrestrial species. We suggest that the interactions among the outputs from the circadian and circatidal clocks have the potential to explain the behaviour of many animals for which the ability to predict semilunar and lunar changes in environmental cues may be extremely important.

## Materials and Methods

Adult *Scyphax* were collected from North Piha Beach on the west coast of New Zealand (36°57′S, 174°28′E) and transported directly to environmental cabinets containing 50 actographs isolated from uncontrolled external vibration. Actographs (100 mm diameter) were filled with sand to a depth of 200 mm into which *Scyphax* burrowed readily. Infrared light gates recorded foraging behaviour across the surface of the sand. Activity was measured by the number of times the light beam was crossed, recorded using ClockLab Software (Actimetrics, IL) every minute for roughly 50 solar days. Temperature was kept at 19 °C ± 1 °C. Animals were fed before but not during experiments. Fifty adult *Scyphax* were recorded in each of six experiments where different lighting and tidal signals were given, summarized in Table 1. White light intensity was 1000 lux during the light phase at the surface of the sand. In the light T-cycle experiments, tidal cycles were simulated by two-hour periods of vibration starting 12.42 hours apart provided by two electric motors. In Experiment 1 activity was recorded in constant darkness (DD). Experiments 2–4 were conducted in light dark (LD) cycles of equal light and dark. In experiments 5 and 6 the light cycles were kept constant (LD12:12) and the tidal T-cycle was manipulated from 12.42 h to 12.25 h and 12.65 h respectively.

### Statistical analysis

The standard chi-squared periodogram technique was used to analyse periodicity in the data at the circadian and circatidal scales (Figure S1). However time series techniques such as the periodogram are not suitable for analysing longer period rhythms, because even with experiments lasting 50 days, there are too few cycles to draw inference with statistical confidence. Instead we developed a novel analytic technique to study the semilunar foraging rhythm. We analysed nightly activity for each animal at the onset of the dark phase for the DD, T24, T24.3, and Tidal T-cycle experiments, and at the conclusion of the dark phase for the T23 experiment, using an activity window of 120 minutes each night (180 minutes for the free-running DD animals). Activity was recorded at the end of the dark phase for the T23 experiment because the shortened T-cycle interferes with activity at the onset of the nightly dark phase. Animals with at least 15 nights of activity in the window were included in the analysis, and were scaled to contribute equally to the analysis. The data constitute the trace of total activity, pooled across animals, for each night in the experiment.

To each activity trace, we fitted a periodic spline curve with period predicted by the beat hypothesis (Equation 1), and recorded the *R* ^*2*^ goodness-of-fit statistic. We used randomization to test the null hypothesis that the fit with this period was no better than chance, by randomly permuting the assignment of activity to days for all animals. We explored three randomization schemes (S3). Scheme 1 permuted the days for all animals independently, to test whether an apparent periodic signal could arise purely by chance, and a significant result from this test was considered the primary evidence for periodic behaviour. Scheme 2 generated a single permutation of days for each randomization and applied it to all animals, to test whether an apparent periodic signal could arise from non-rhythmic effects common to all animals. Scheme 3 tested whether animals were in phase with each other by using a random start day for each animal but thereafter maintaining the correct day order. A significant result from Scheme 3 would supply evidence of a common phasing. The *R* ^*2*^ statistic was calculated for each randomization, and the proportion of random fits superior to the real-data fit supplied the *P*-value.

## Electronic supplementary material

Supplementary Information
