# Supplementary material for: Circadian and circatidal clocks control the mechanism of semilunar foraging behaviour
Source: Sci Rep. 2017 Jun 19;7:3780. doi: 10.1038/s41598-017-03245-3 (PMC5476643; doi:10.1038/s41598-017-03245-3)
Supplement: Supplementary file 1 — Supplementary Information [file 41598_2017_3245_MOESM1_ESM.pdf]

Circadian and circatidal clocks control the mechanism of semilunar foraging behaviour

Supplementary Information

Authors: James F. Cheeseman, Rachel M. Fewster, Michael M. Walker.

Figure S1. (Page 3)
Periodogram analysis of the shorter period circatidal and circadian rhythms in the combined data of each experiment (1) Constant darkness, n = 20; (2) T=24 hours, n = 25; (3) T=23 hours, n = 20; (4) T=24.3 hours, n = 18; (5) Tidal T =12.25 hours, n = 19; (6) Tidal T =12.65 hours, n = 20 (see also main figure 1).

Figure S2. (Pages 4-21)
Supplementary data: Individual actograms of all animals used in the analysis for each experiment: Experiment 1 Constant darkness, Experiment 2 T=24, Experiment 3 T=23, Experiment 4 T=24.3, Experiment 5 T_t=12.25 T=24, Experiment 6 T_t=12.65 T=24

Figure S3. (Pages 22-39)
Randomisation results and example model fits. Results are shown for randomisation schemes 1, 2, and 3, for each of experiments 1 to 6. Experiment name and details are printed at the bottom left of each page. The first panel on each page shows the fit to the real data: black lines indicate the observed data of *Scyphax* activity in the analysis window per night; red curves show the model fit with the beats-predicted period. Grey bars represent nights when the tidal signal coincided with the analysis window; heights of bars indicate the duration of coincidence. Curves are fitted to the data using penalised least-squares (LS); details above the plots show the R^2 statistic for the fit, as well as the attained minimum (Min) and least-squares component (LS); the difference between Min and LS is due to shape penalties imposed on the periodic spline curves; the observed small differences indicate that the fitted curves successfully attained the shape requirements. *Experiment-days* correspond to light-dark cycles as perceived by the animals; for example in experiment 3 (T23), each experiment-day is 23 hours long. Each page shows four other panels with black lines and fitted blue curves; these constitute four examples of randomised data and model fits under the specified randomisation scheme. R^2 statistics from the blue curves create the null distribution for each hypothesis test. Histograms in the bottom left panels show R^2 results from the null distribution, created from 500 randomisations including the four shown. The vertical red line shows the R^2 obtained from the real-data fit; the corresponding *p*-value for the randomisation test is printed above the box.

Figure S4. (Pages 40-45)

R^2 results from periodic spline models fitted to the observed data of *Scyphax* activity from experiments 1 to 6 as the hypothesised period is varied from 5.0 to 26.0 in steps of 0.1 experiment-days. Experiment name and details are printed at the bottom left of each page. *Experiment-days* correspond to light-dark cycles as perceived by the animals; e.g. in experiment 3 (T23), each experiment-day is 23 hours long. For each period from 5.0 to 26.0, a periodic spline with this period is fitted to the real data and the resulting R^2 statistic is plotted in the black line. The period corresponding to the beats hypothesis is depicted by the red vertical line and typically coincides with the region of highest R^2 results. The green horizontal line shows the maximum R^2 attained across all periods from 5.0 to 26.0.

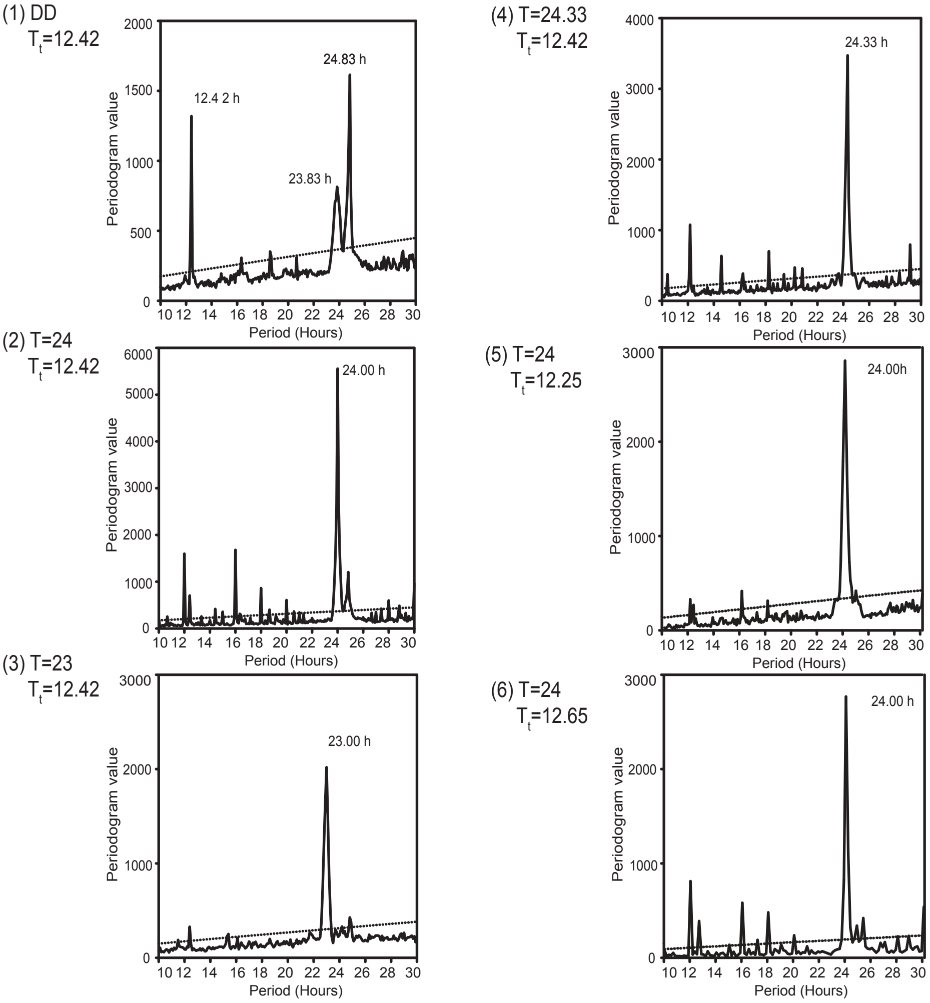
(1) DD
T_t=12.42
12.4 2 h
24.83 h
23.83 h
(2) T=24
T_t=12.42
24.00 h
(3) T=23
T_t=12.42
23.00 h
(4) T=24.33
T_t=12.42
24.33 h
(5) T=24
T_t=12.25
24.00h
(6) T=24
T_t=12.65
24.00 h
Periodogram value
Period (Hours)

(59)

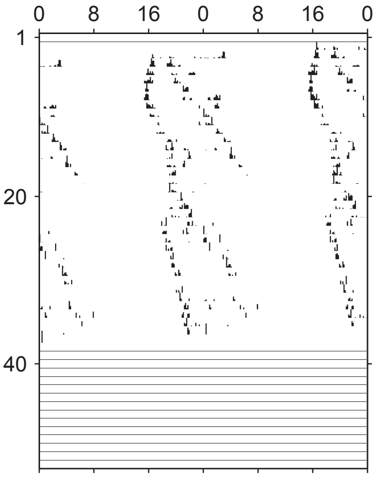

(62)

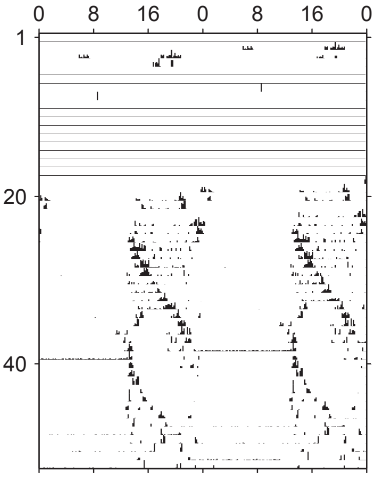

(71)

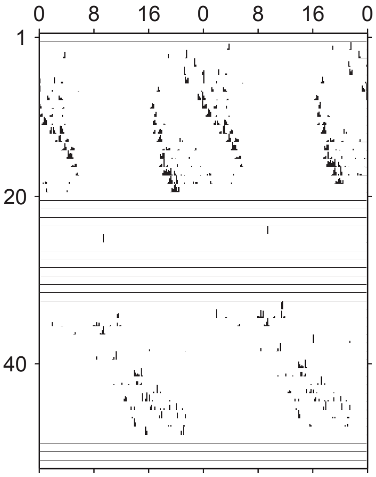

(73)

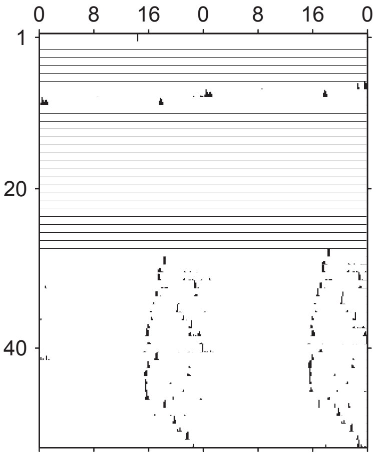

(74)

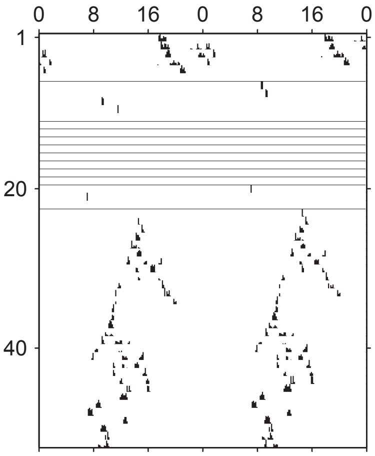

(80)

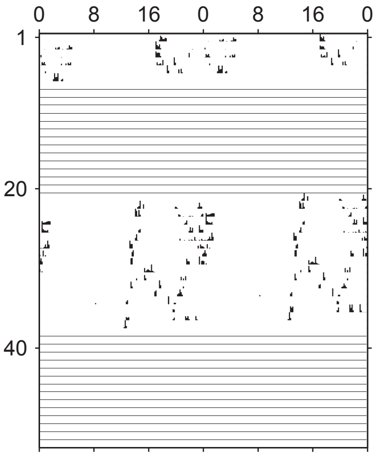

(81)

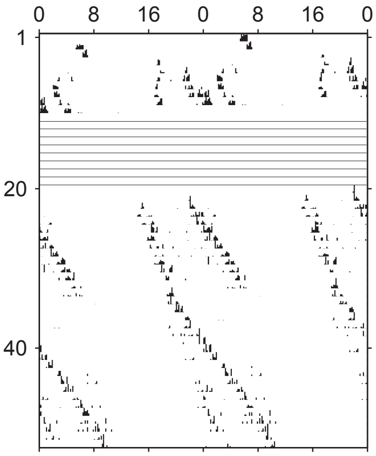

(86)

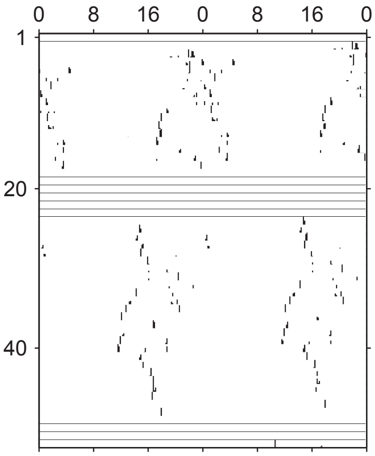

(88)

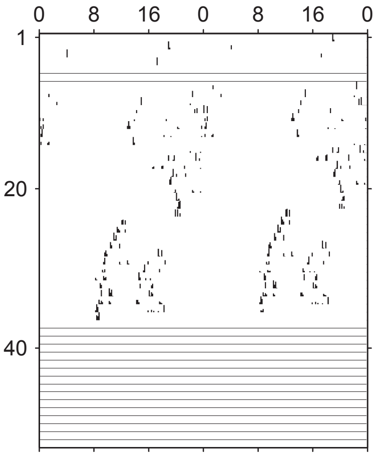

(91)

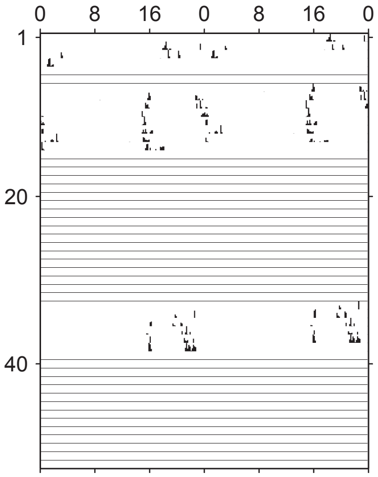

(92)

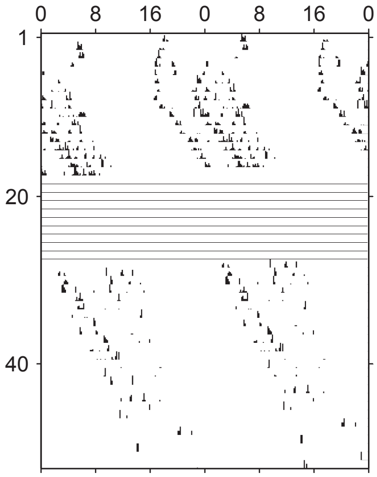

(94)

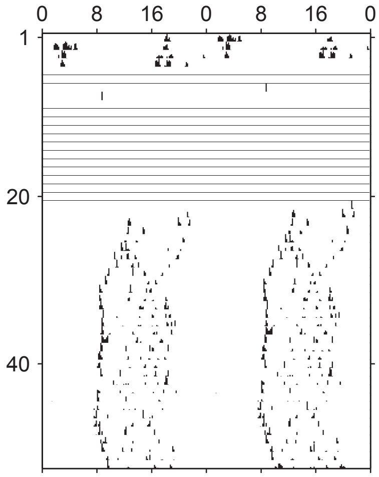

(95)

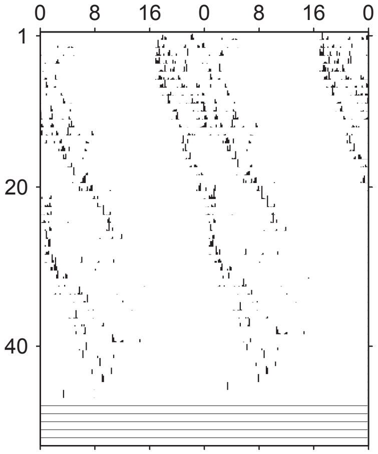

(96)

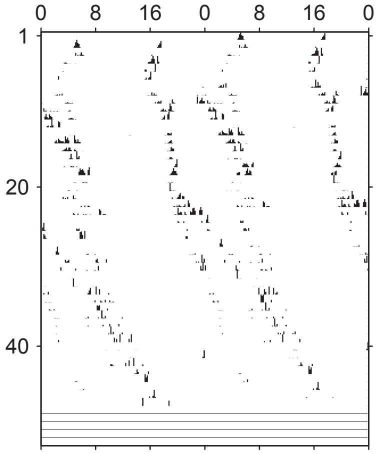

(97)

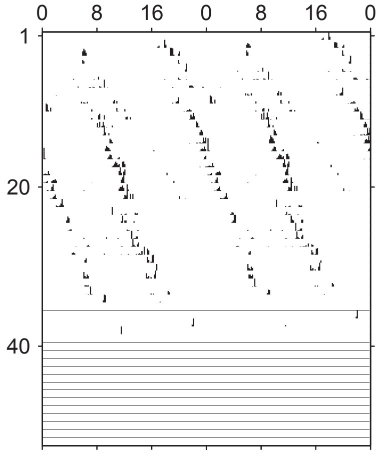

(99)

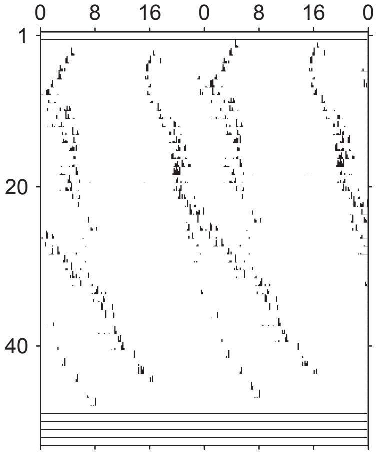

(100)

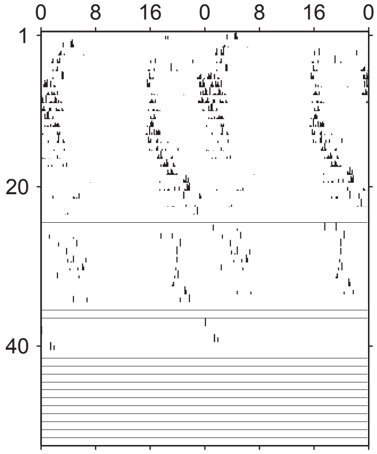

(102)

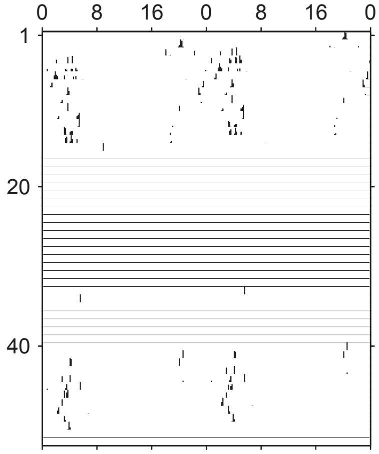

(104)

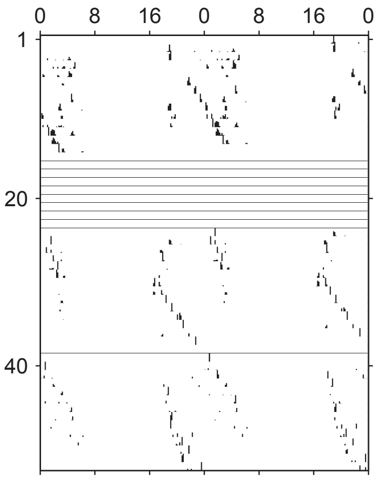

(106)

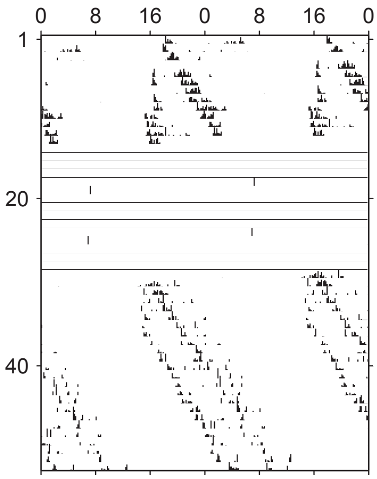

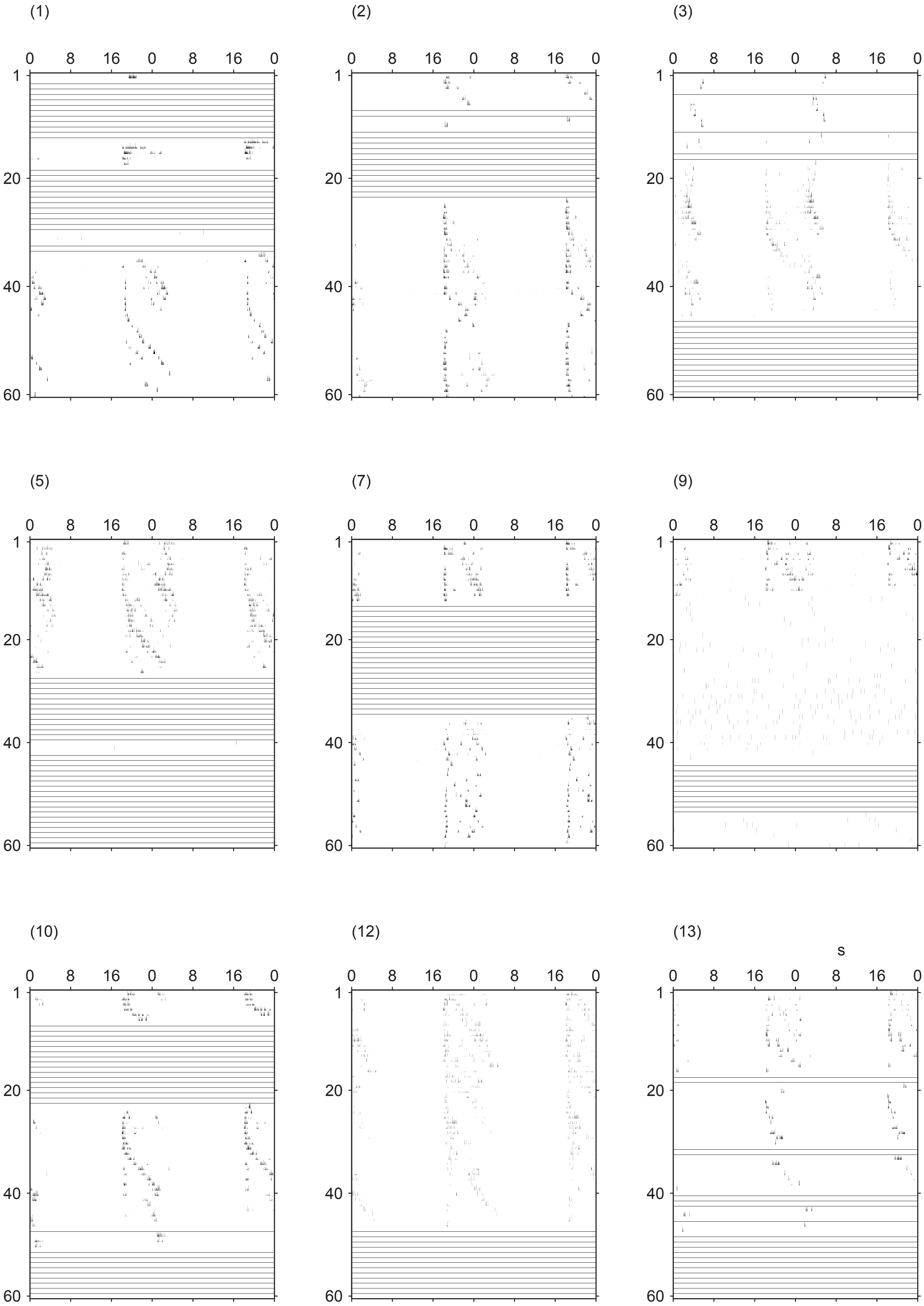
(1)
(2)
(3)
(5)
(7)
(9)
(10)
(12)
(13)
s
0 8 16 0 8 16 0
1 20 40 60

(14)

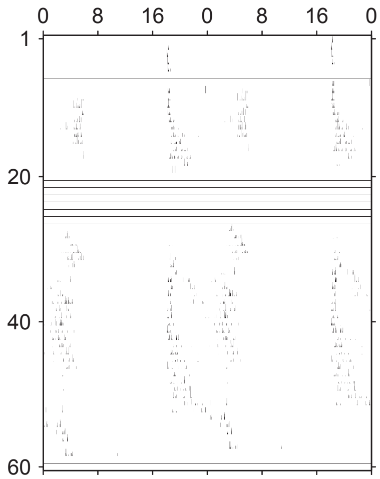

(15)

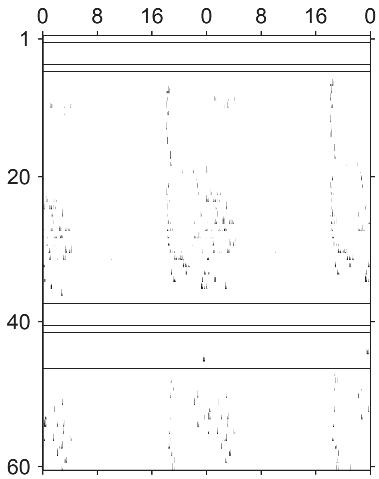

(16)

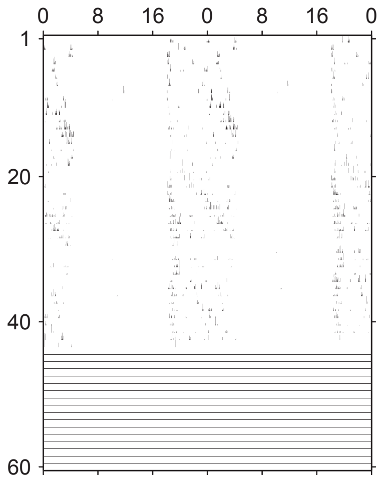

(19)

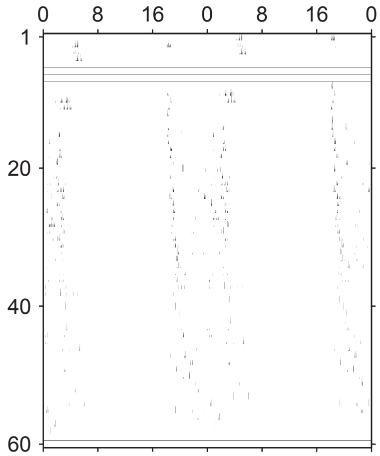

T24026 (29)

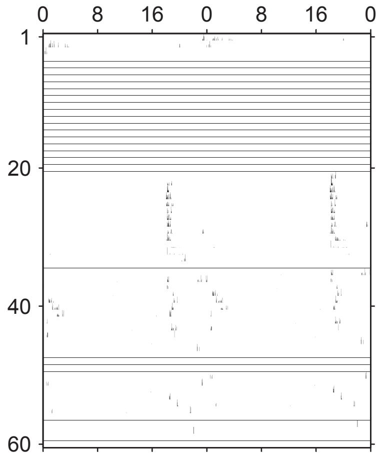

(31)

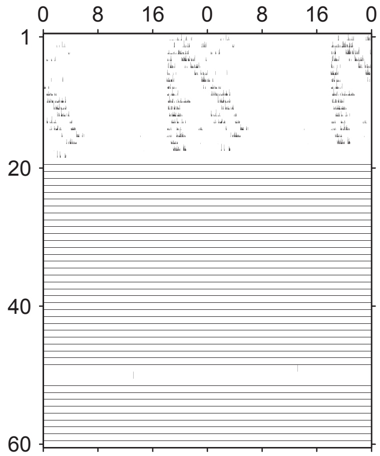

(38)

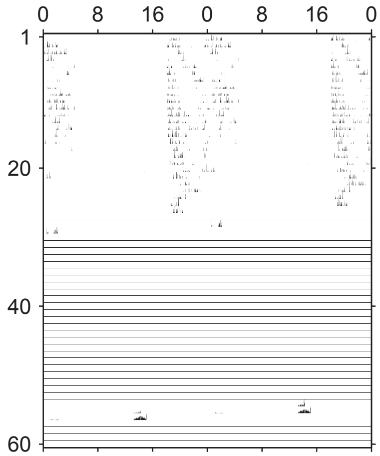

(42)

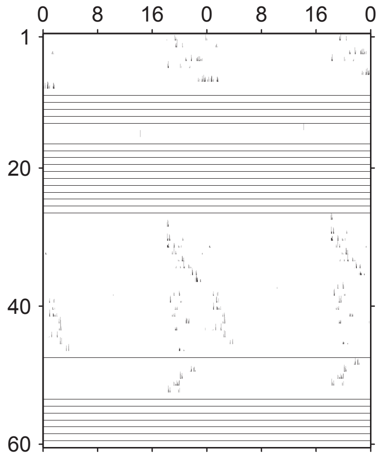

(43)

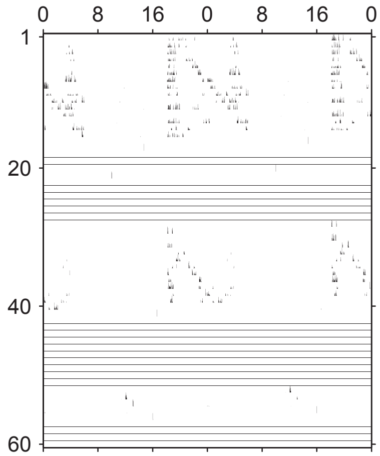

(45)

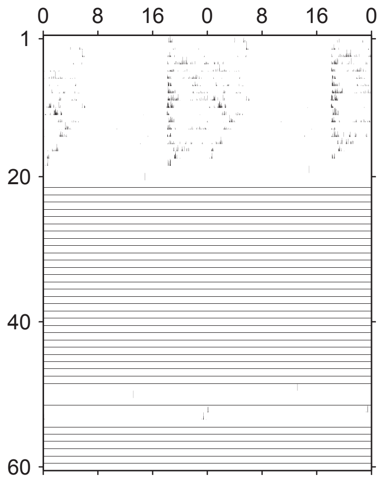

(46)

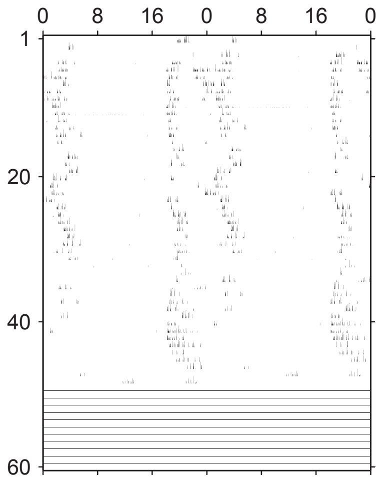

(47)

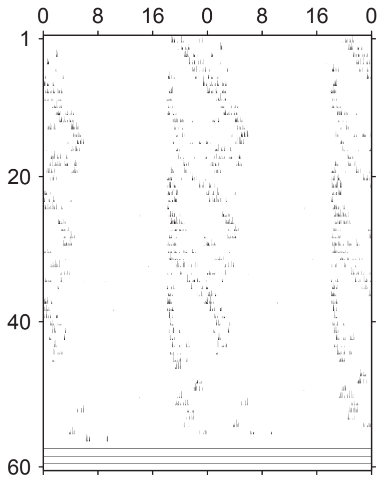

(48)

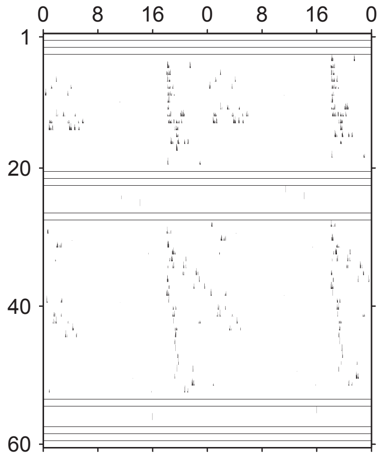

(49)

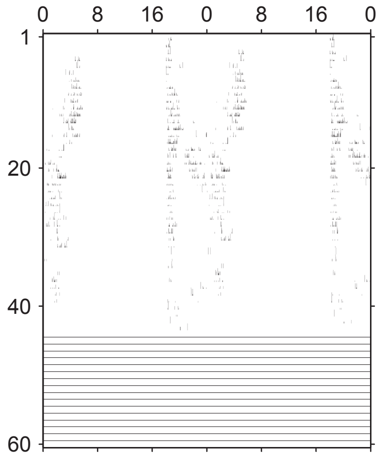

(51)

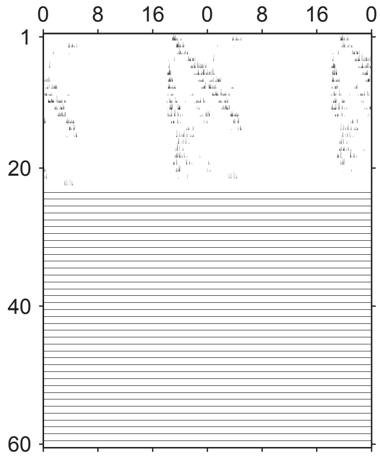

(53)

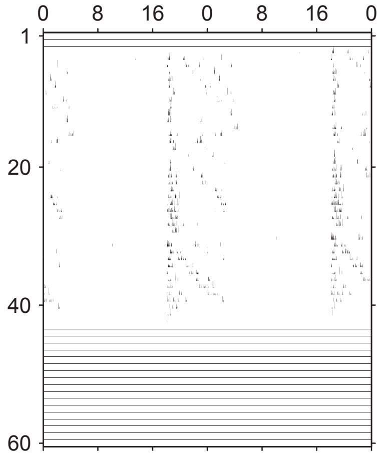

(4)

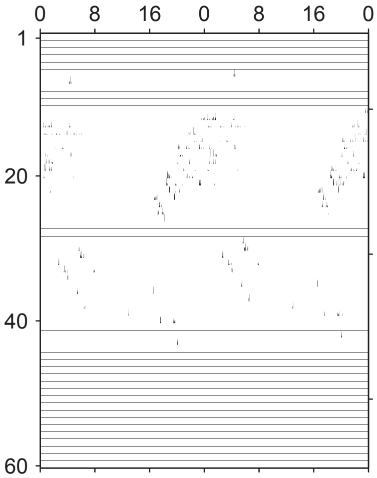

(5)

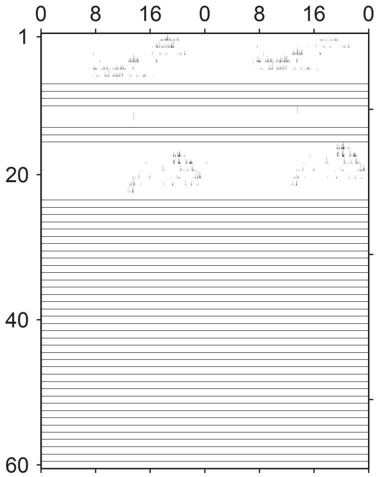

(9)

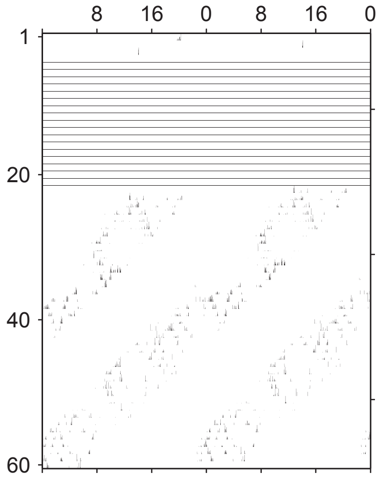

11 (11)

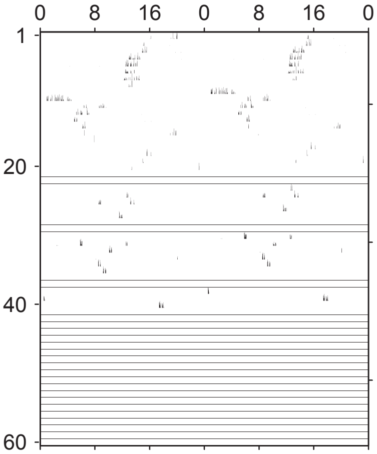

13 (13)

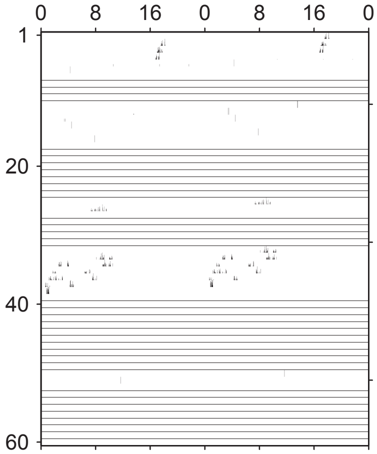

(15)

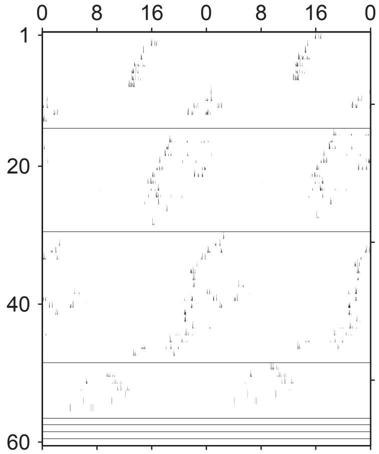

(17)

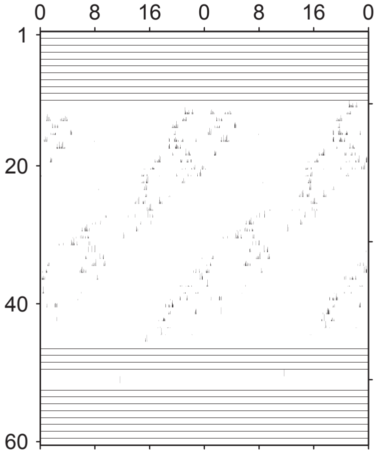

(18)

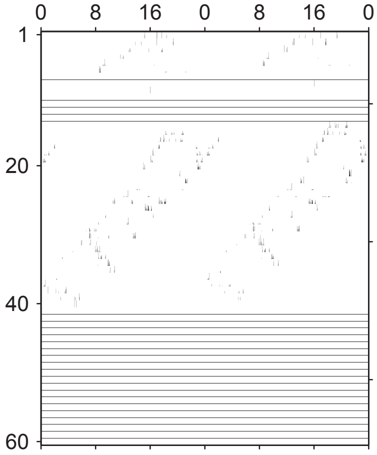

(30)

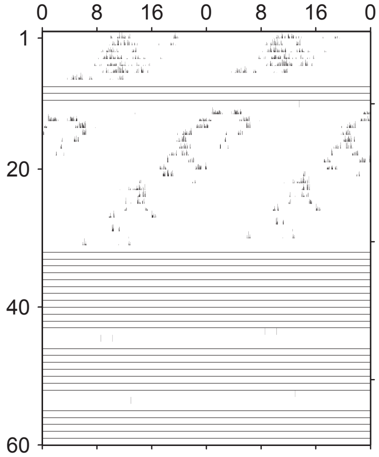

(31)

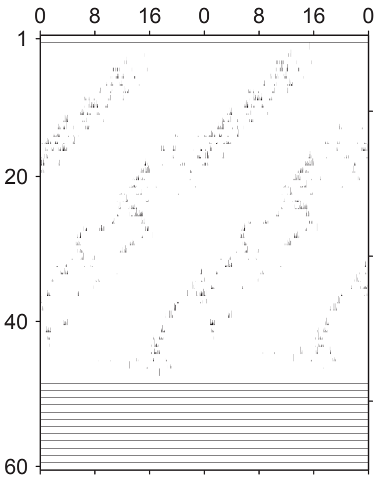

(36)

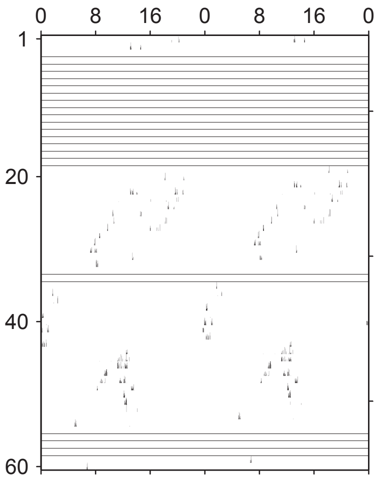

(37)

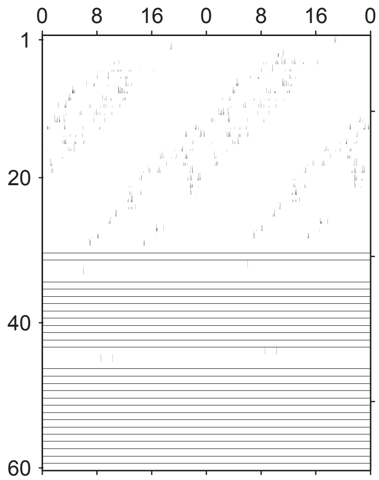

(39)

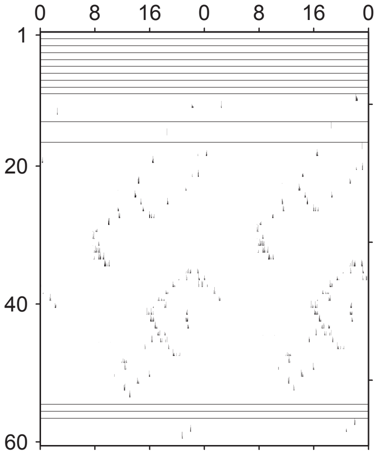

40 (40)

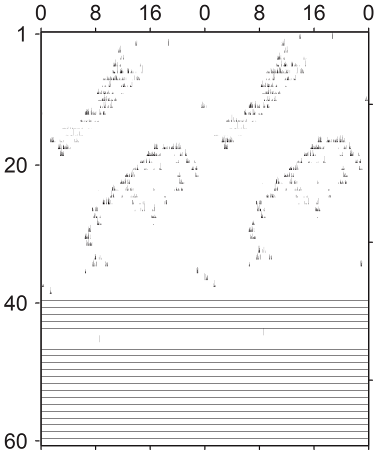

(45)

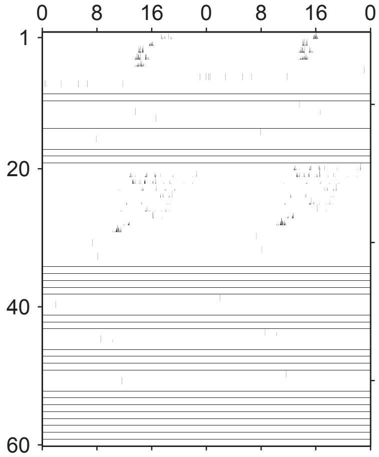

(47)

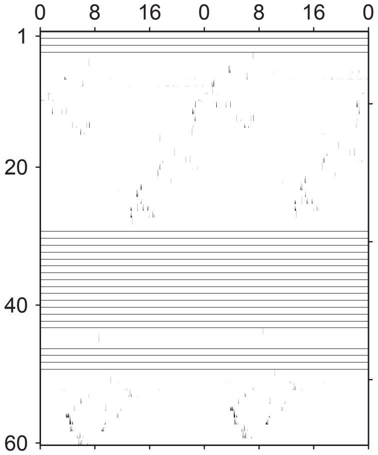

(48)

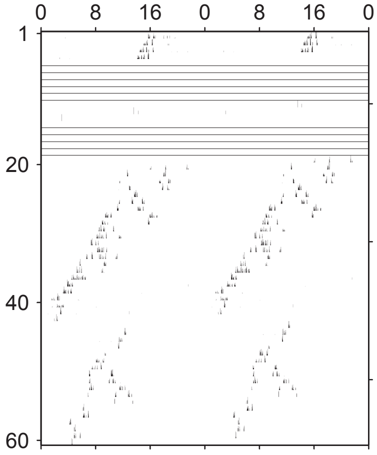

(49)

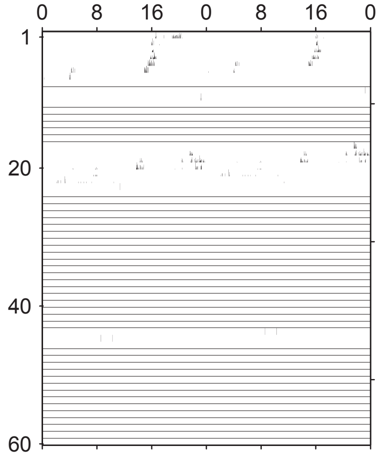

(52)

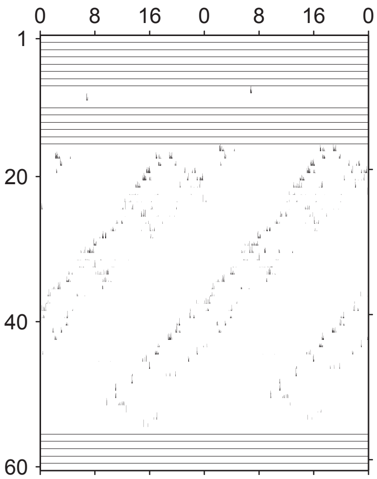

(53)

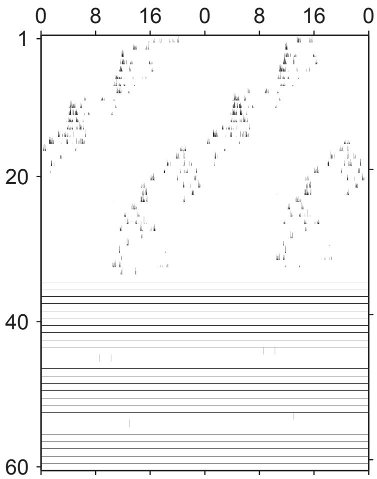

(59)

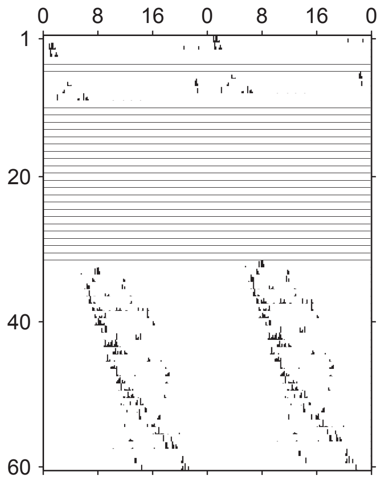

(67)

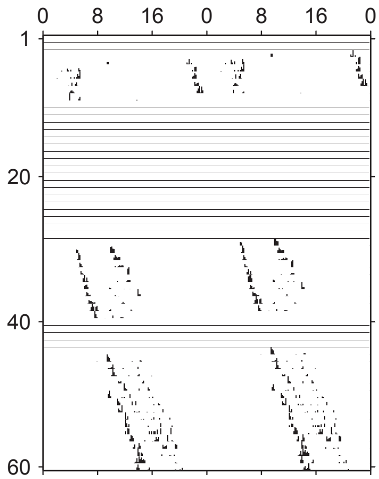

(73)

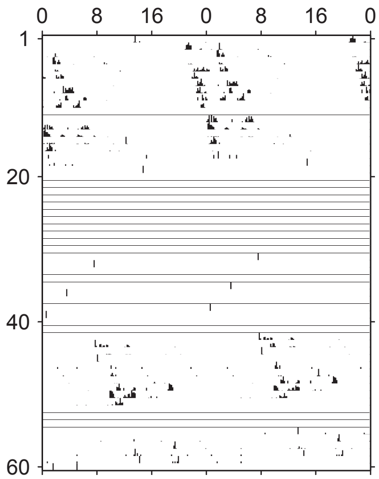

(74)

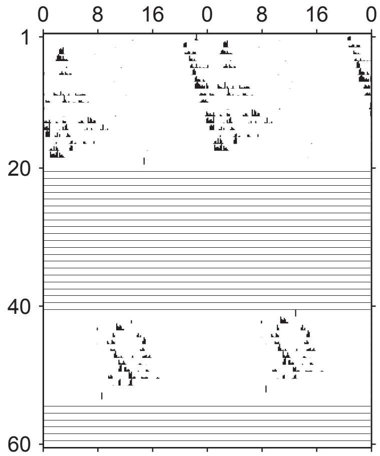

(75)

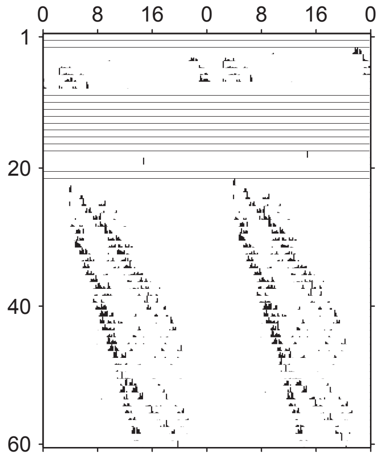

(80)

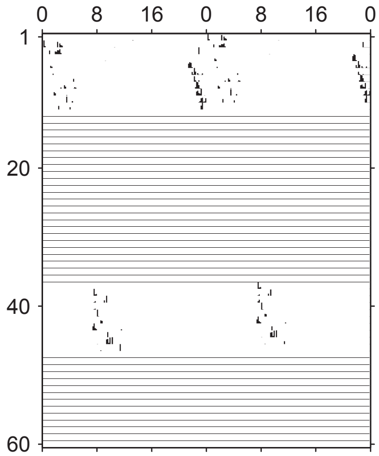

(81)

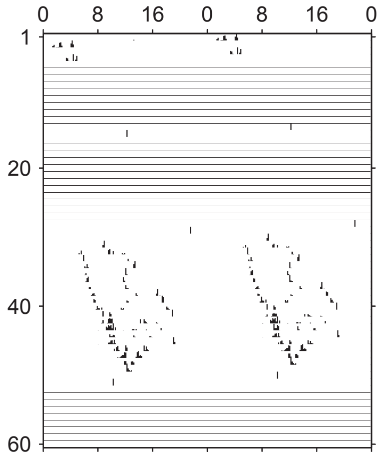

(85)

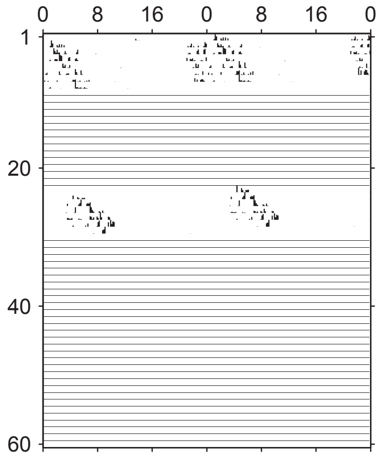

(86)

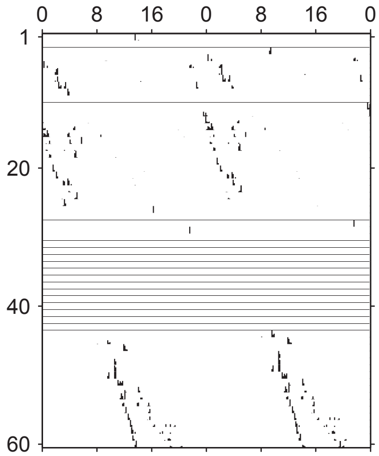

(87)

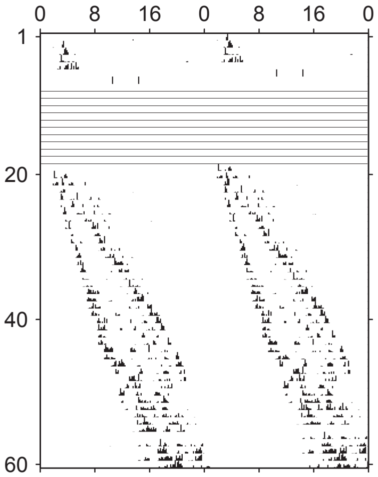

(90)

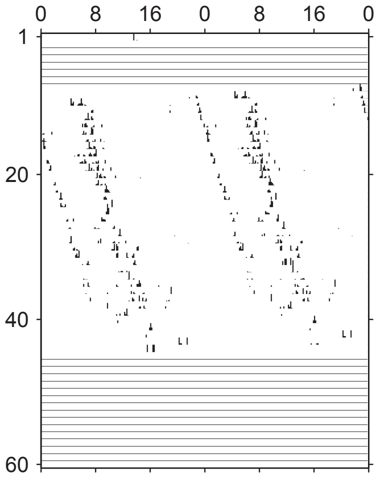

(92)

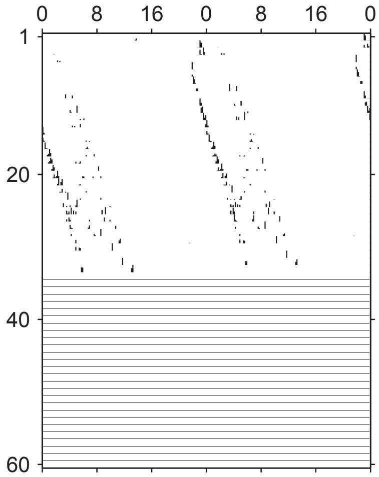

(96)

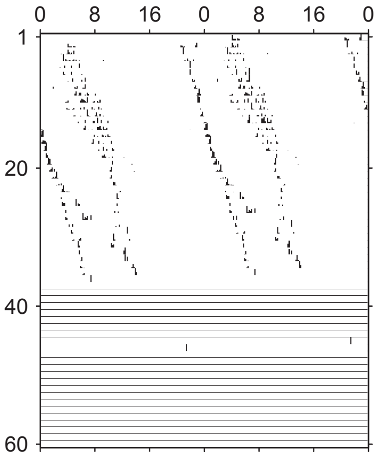

(97)

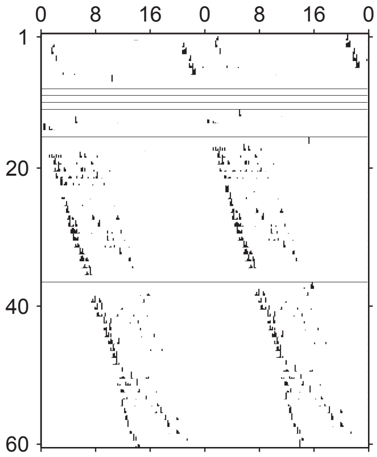

(99)

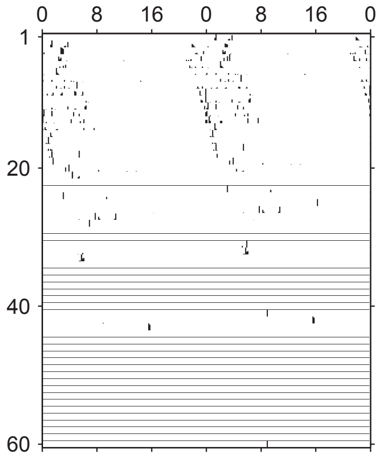

(100)

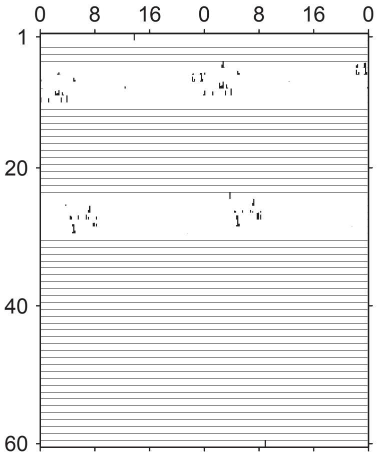

(102)

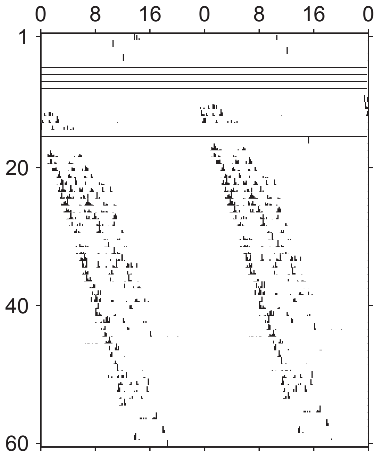

(108)

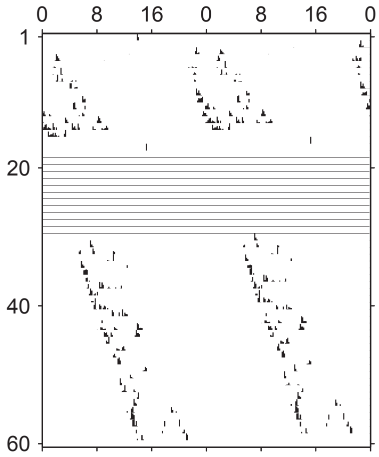

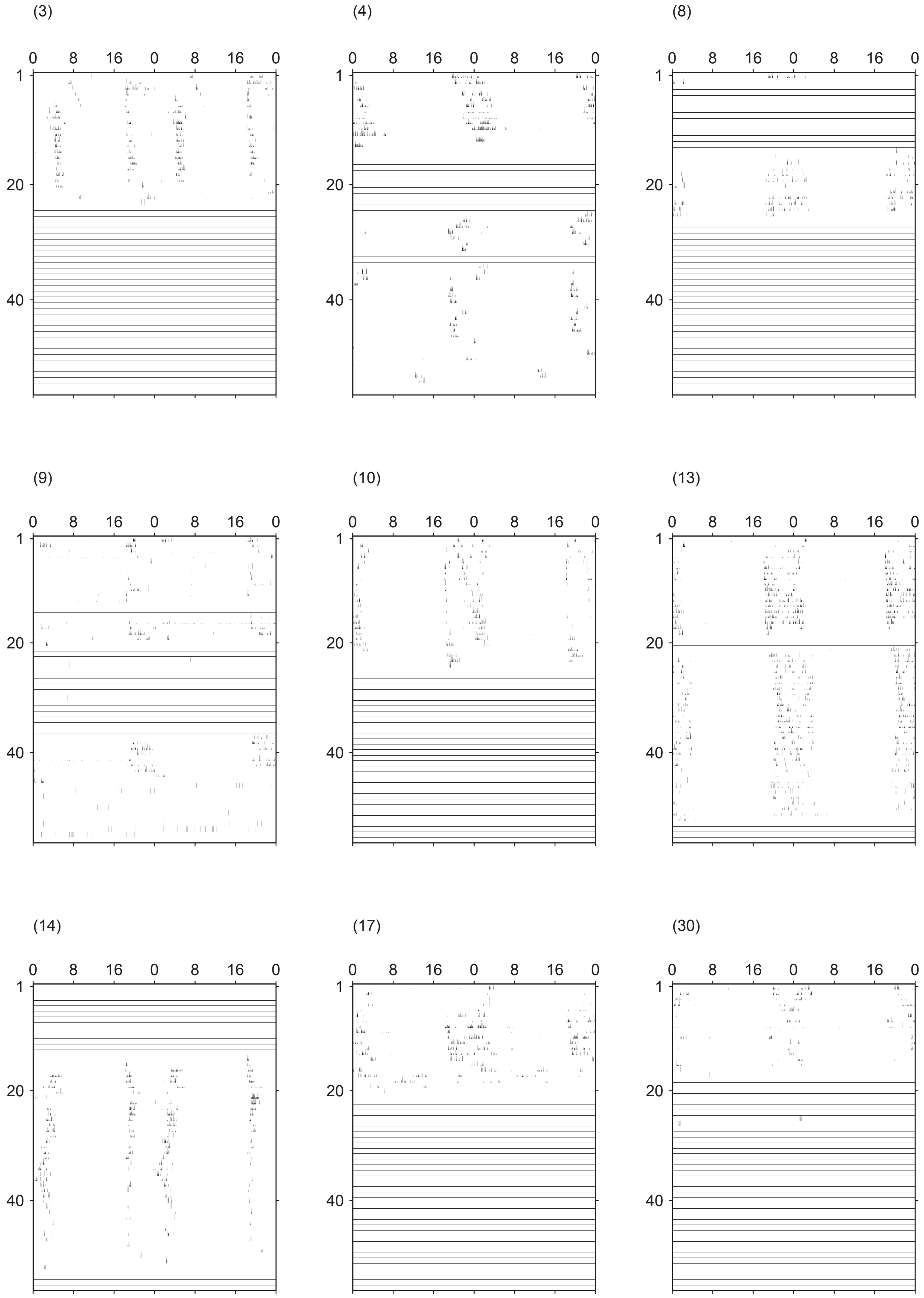
(3)
(4)
(8)
(9)
(10)
(13)
(14)
(17)
(30)
0 8 16 0 8 16 0
1
20
40

(34)

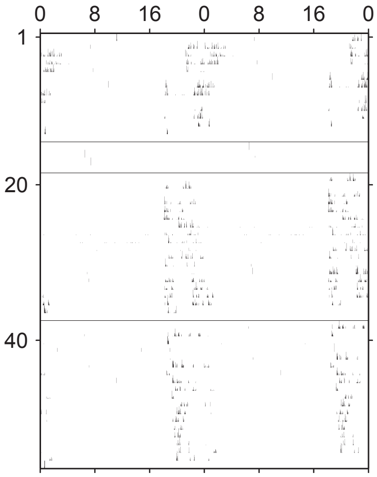

(36)

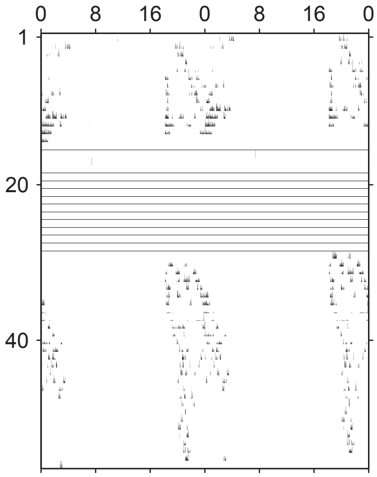

(39)

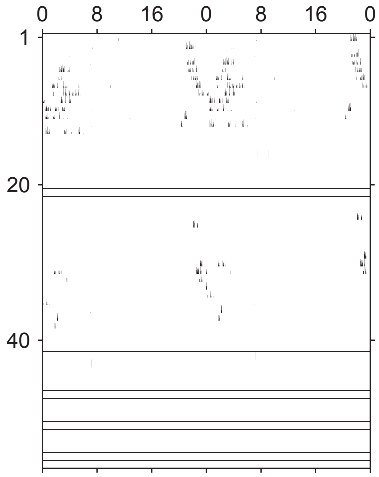

(42)

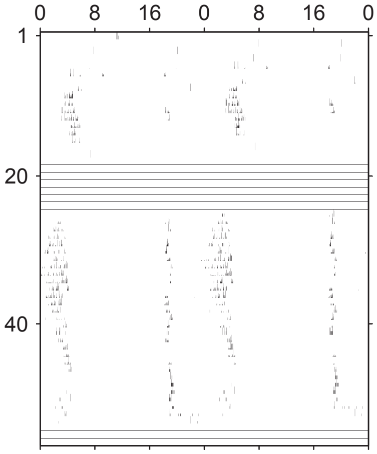

(44)

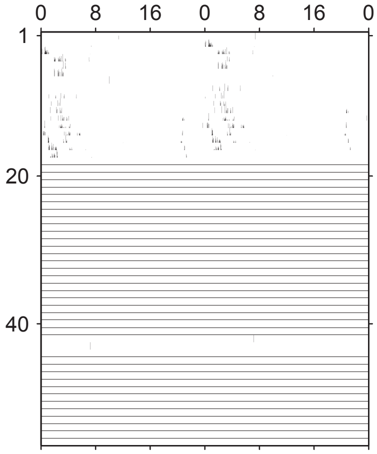

(46)

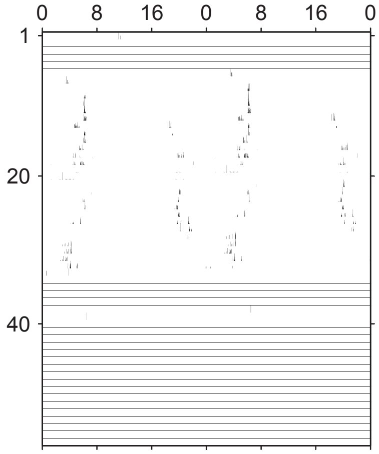

(47)

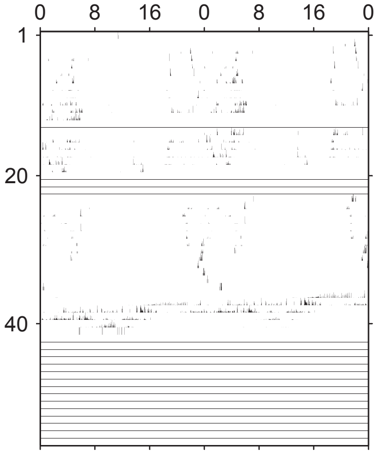

(49)

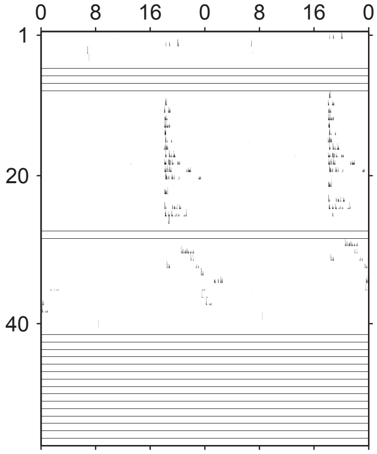

(50)

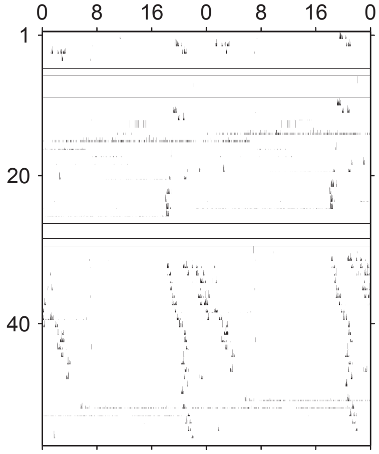

(53)

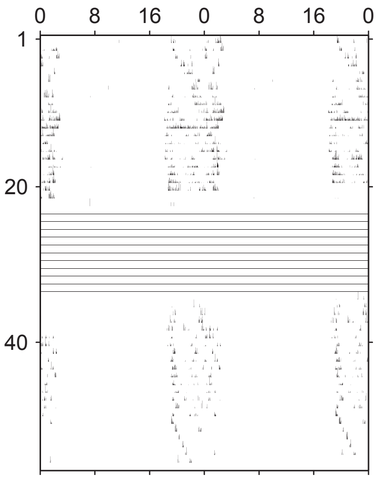

(2)

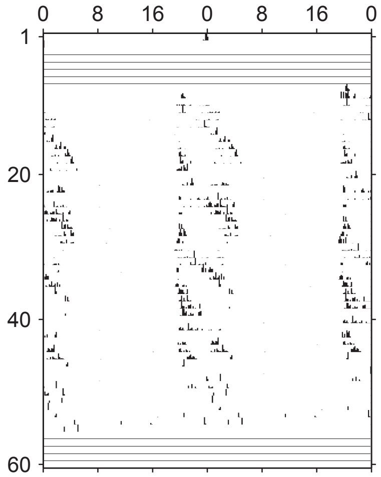

(4)

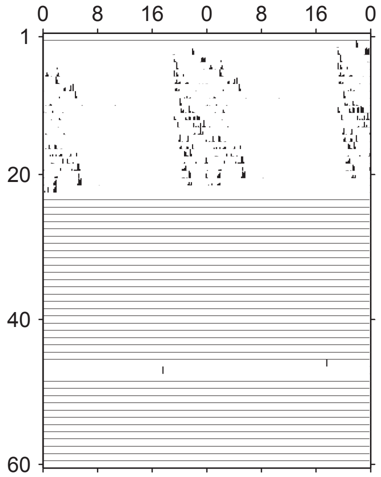

(6)

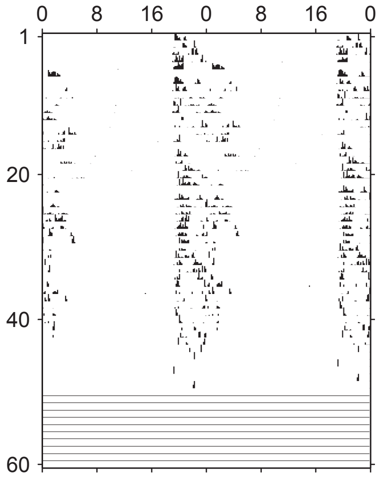

(7)

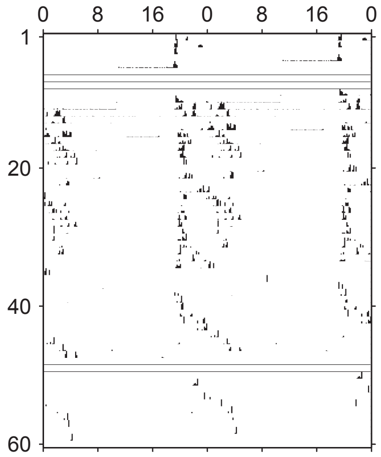

(14)

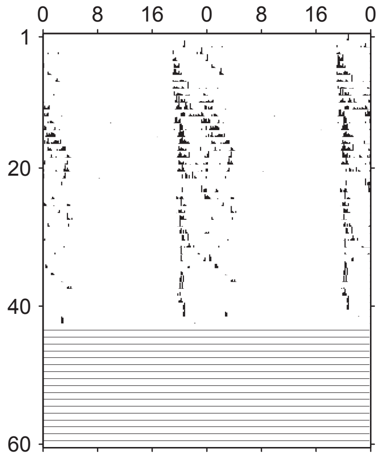

(18)

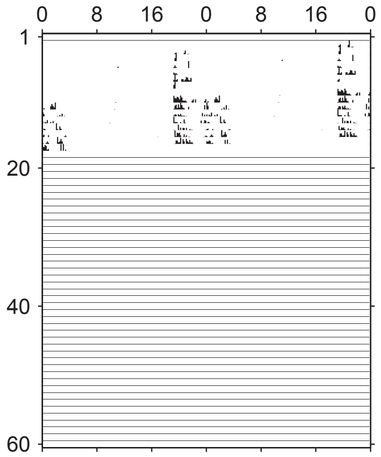

(19)

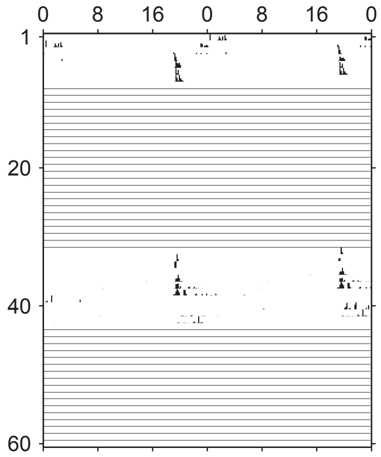

(20)

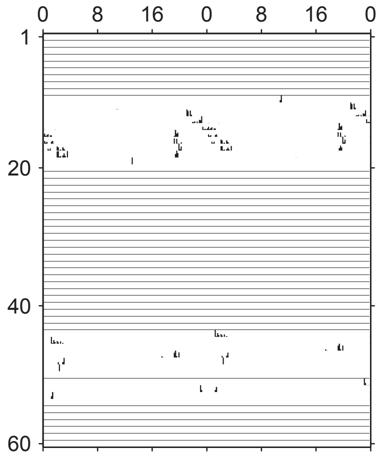

(22)

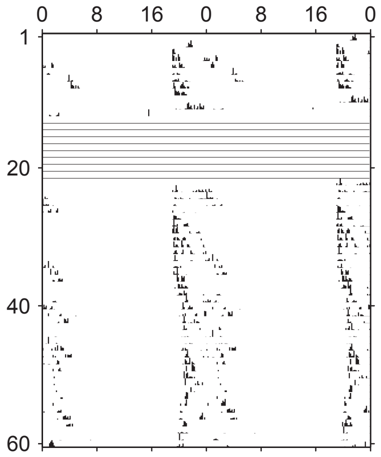

(24)

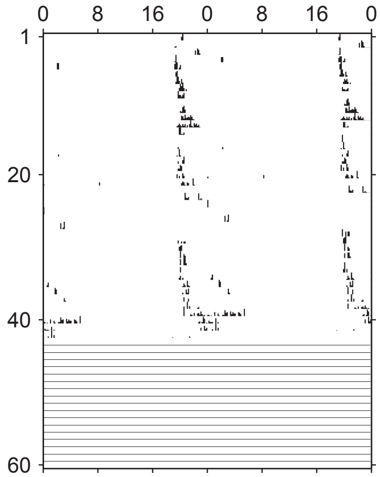

(25)

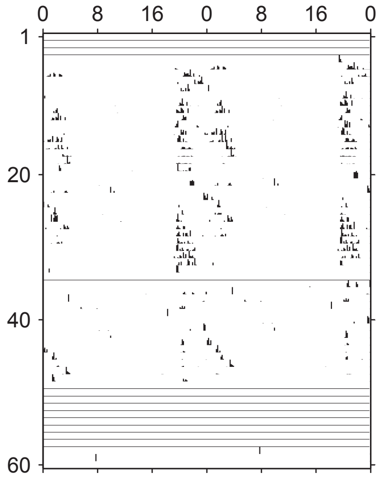

(30)

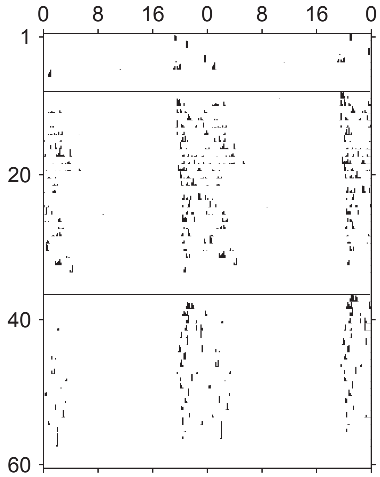

(32)

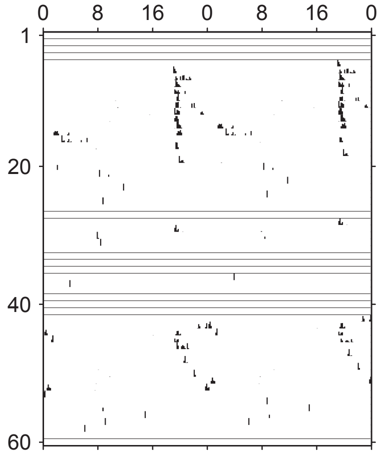

(33)

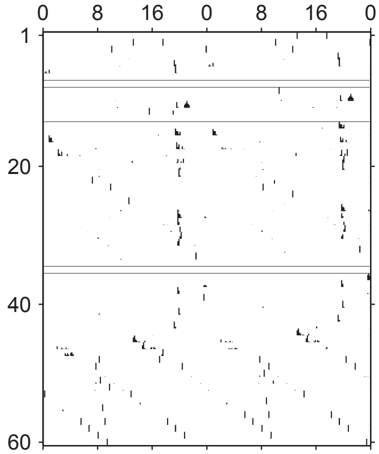

(34)

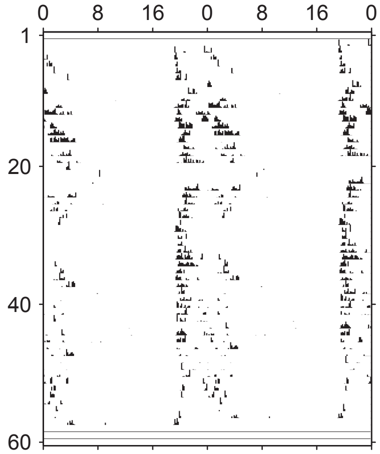

(35)

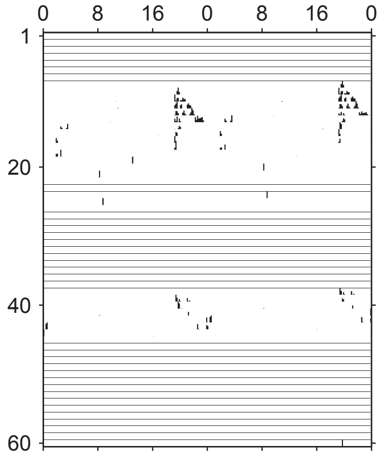

(36)

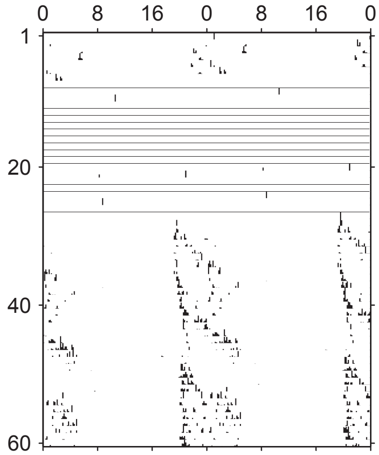

(37)

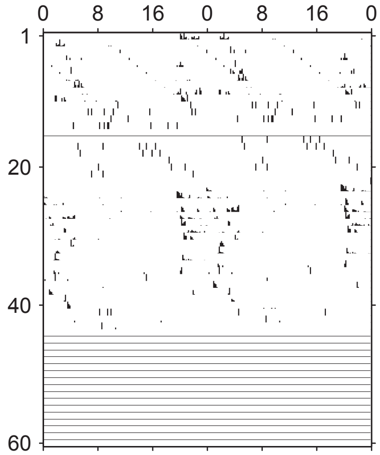

(39)

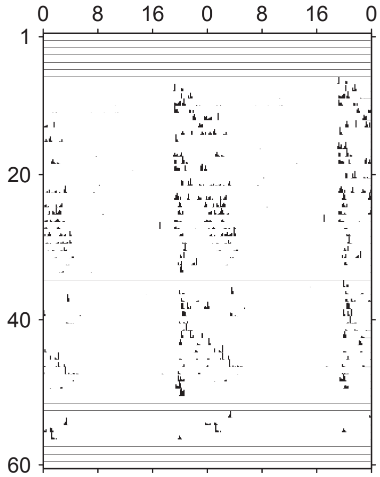

(40)

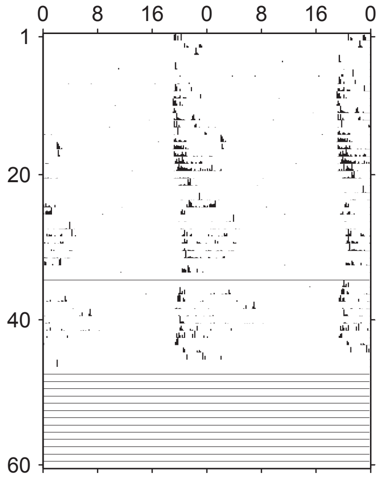

(42)

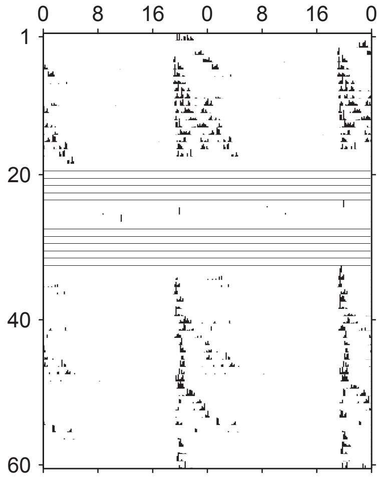

(43)

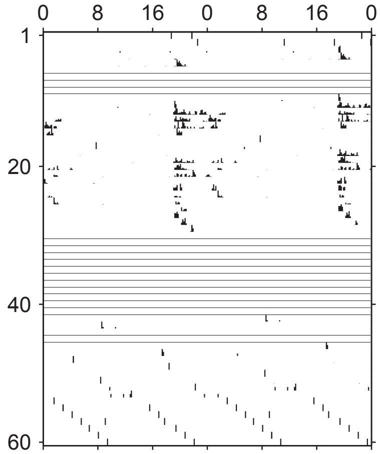

(45)

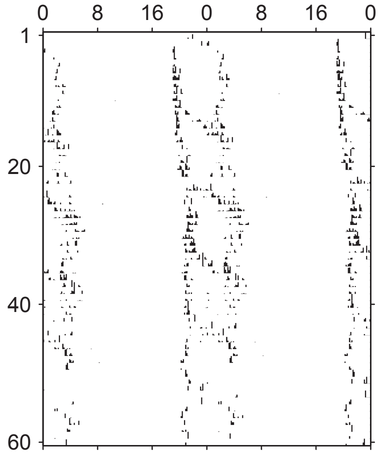

(46)

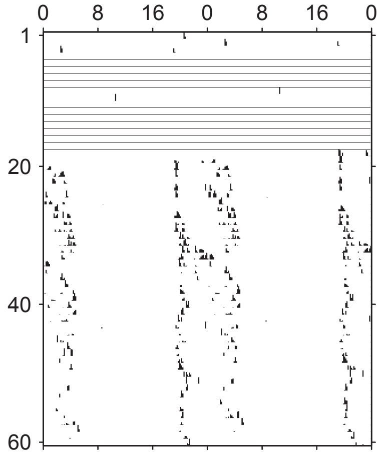

(49)

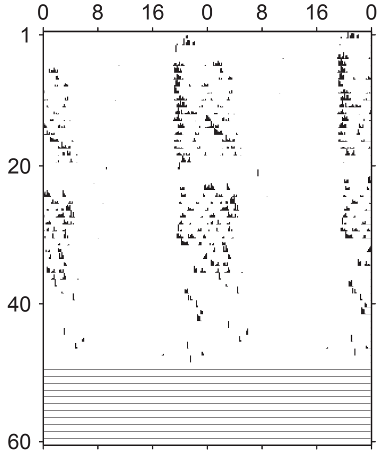

(50)

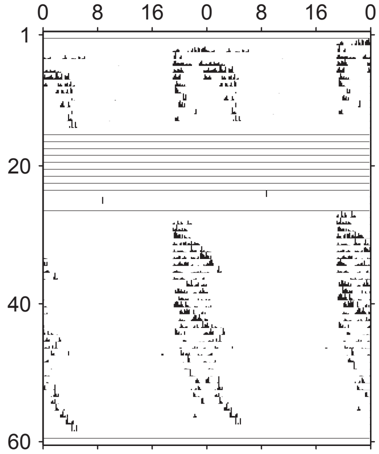

(51)

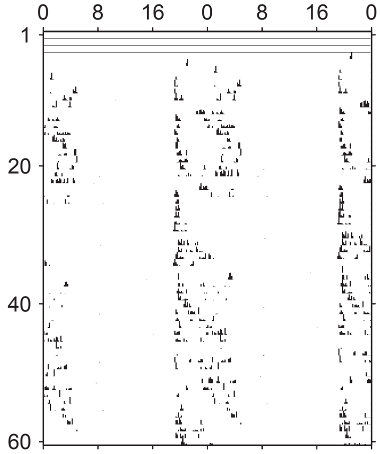

(52)

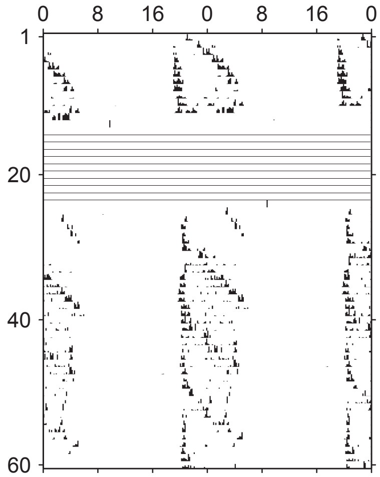

(53)

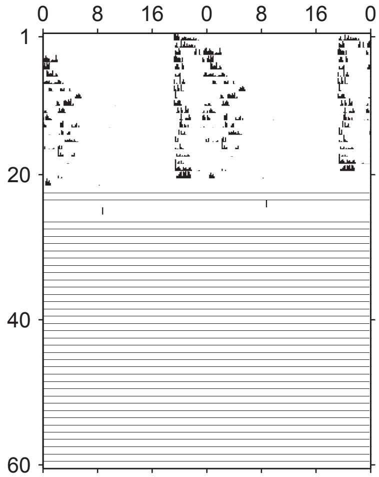

Scheme 1: Days randomised for each animal separately for each simulation

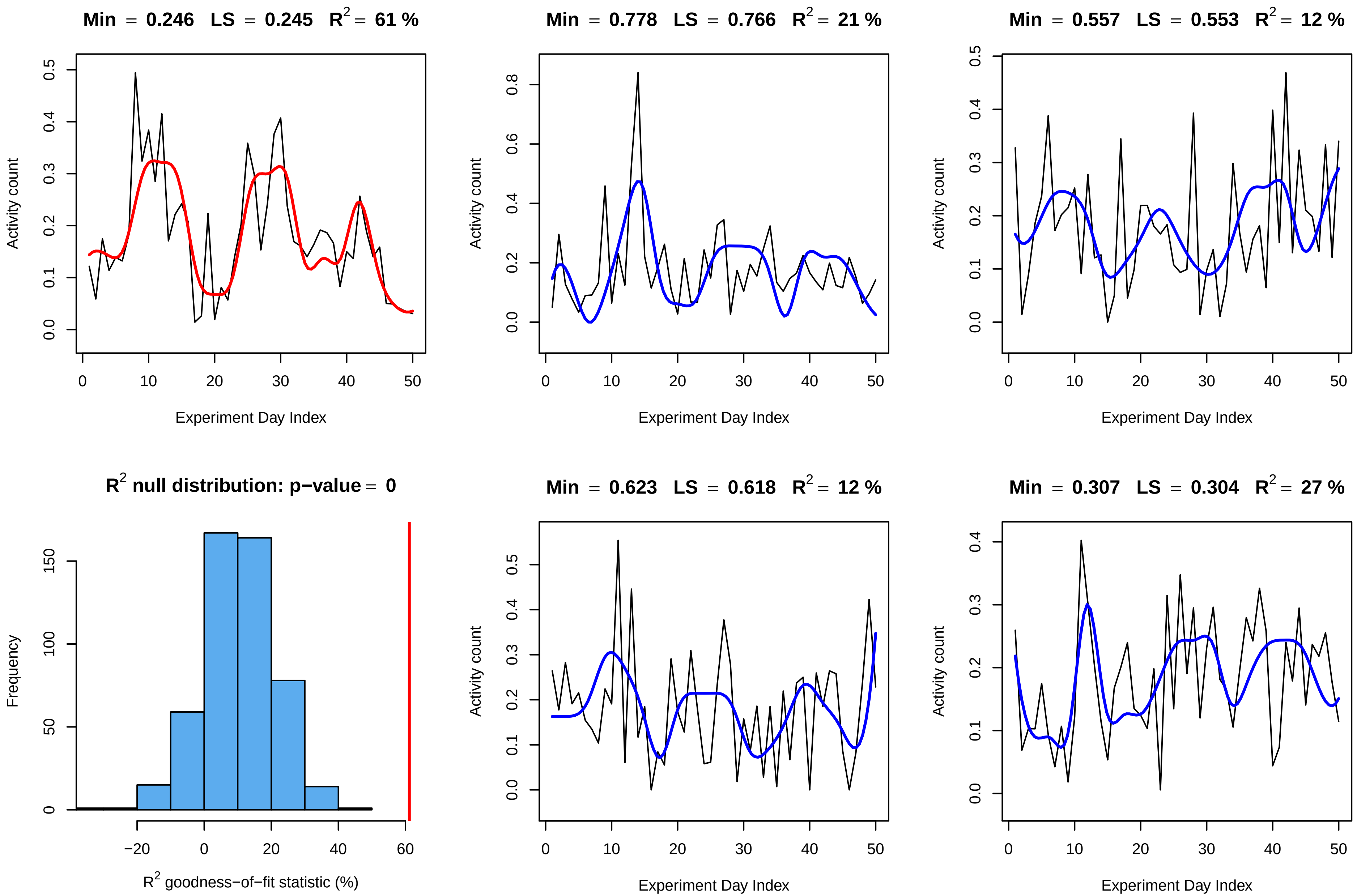

TDARK : res.scheme1.exptdays2.51

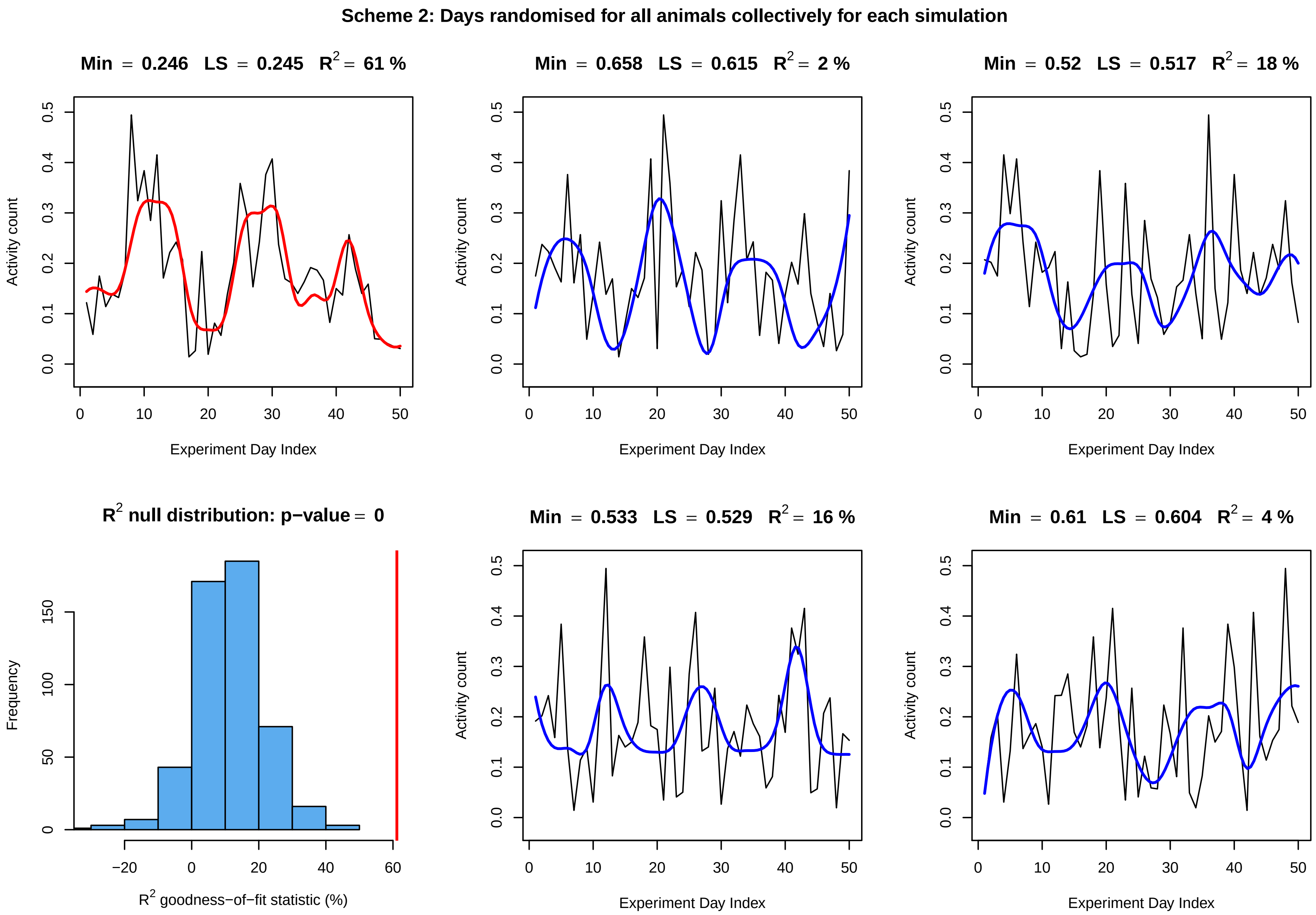

TDARK : res.scheme2.exptdays2.51

Scheme 3: Day order preserved for each animal but with random start−day, selected for each animal separately for each simulation

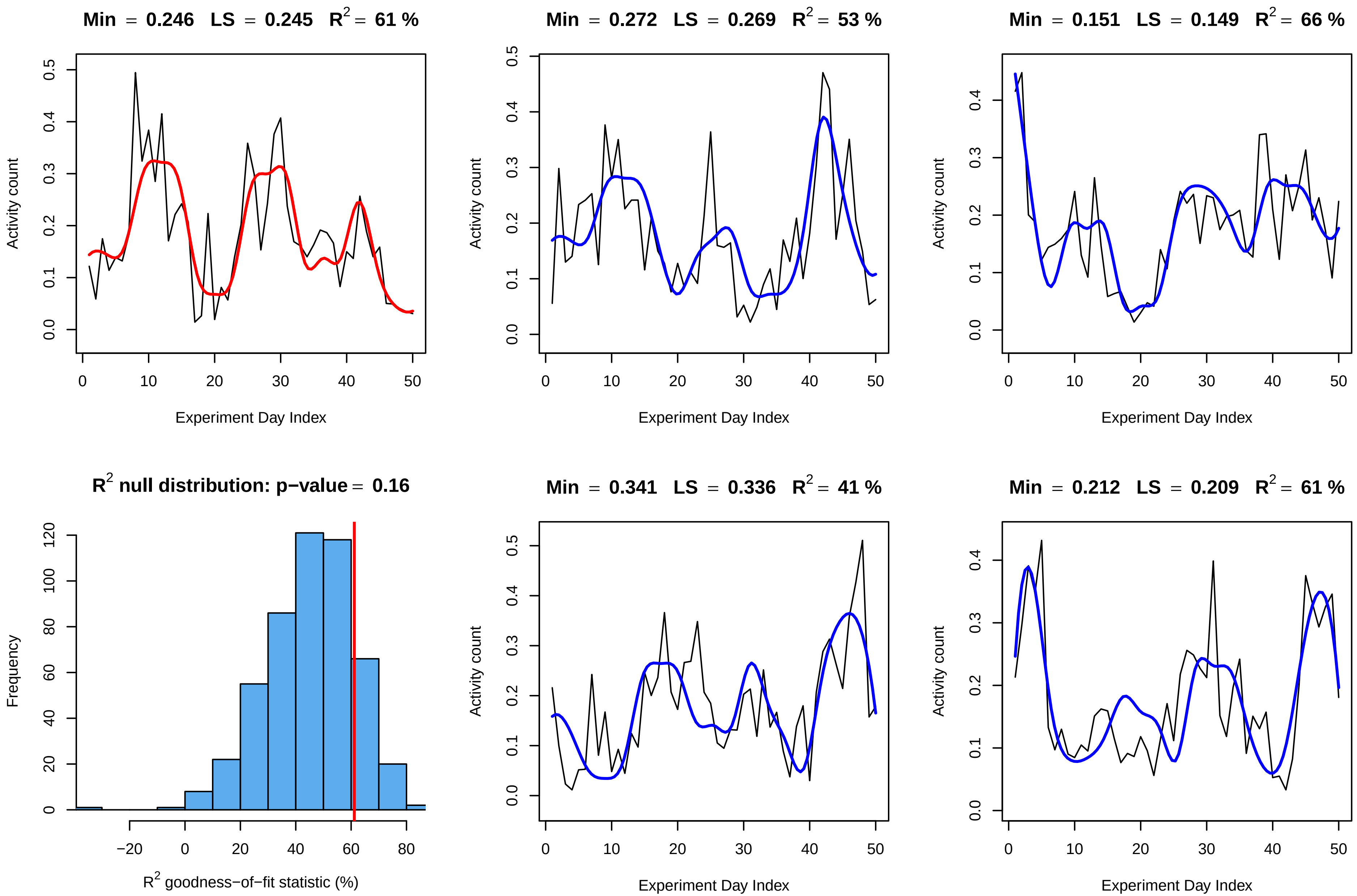

TDARK : res.scheme3.exptdays2.51

Scheme 1: Days randomised for each animal separately for each simulation

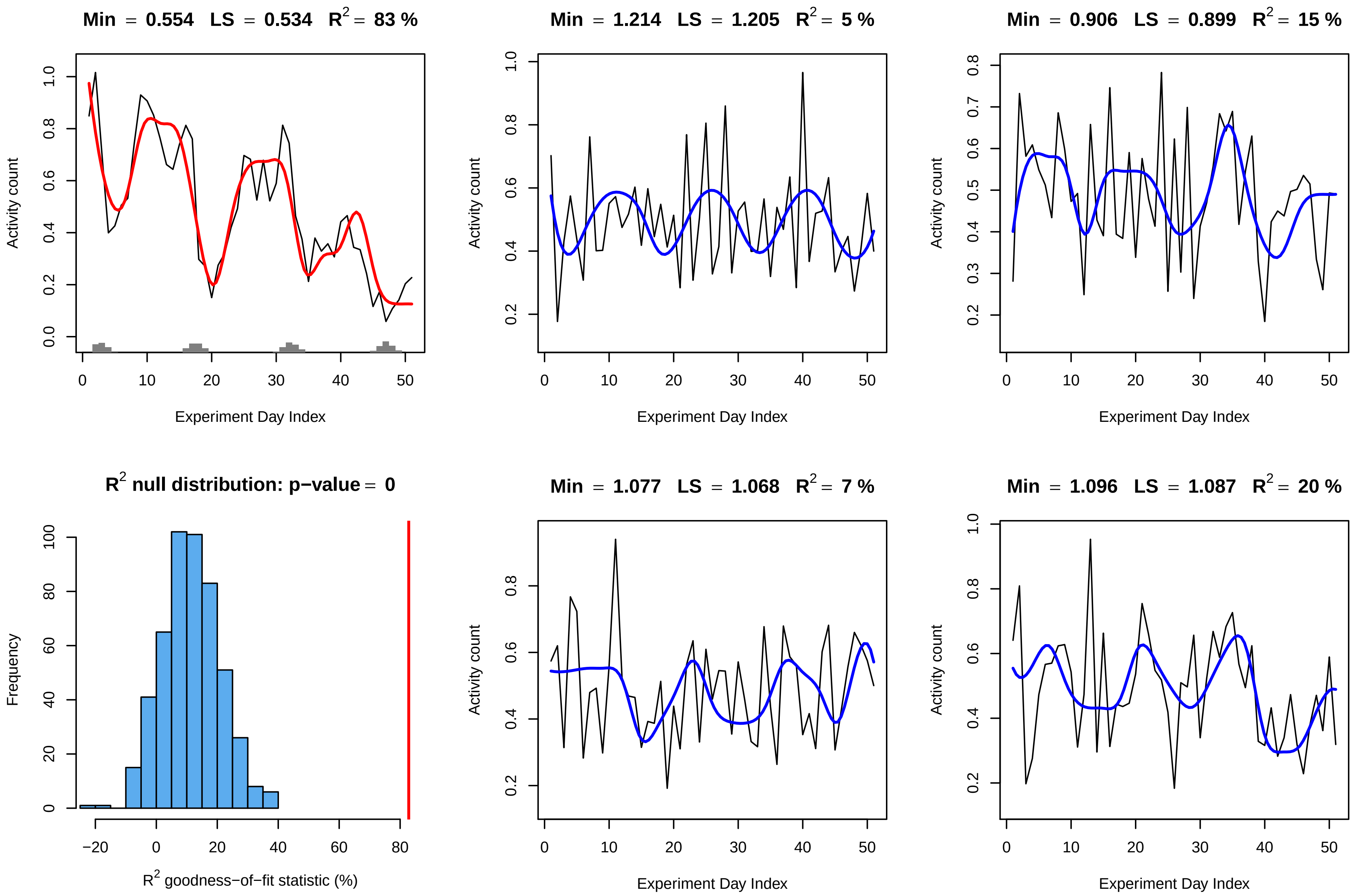

T24 : res.scheme1.exptdays1.51

Scheme 2: Days randomised for all animals collectively for each simulation

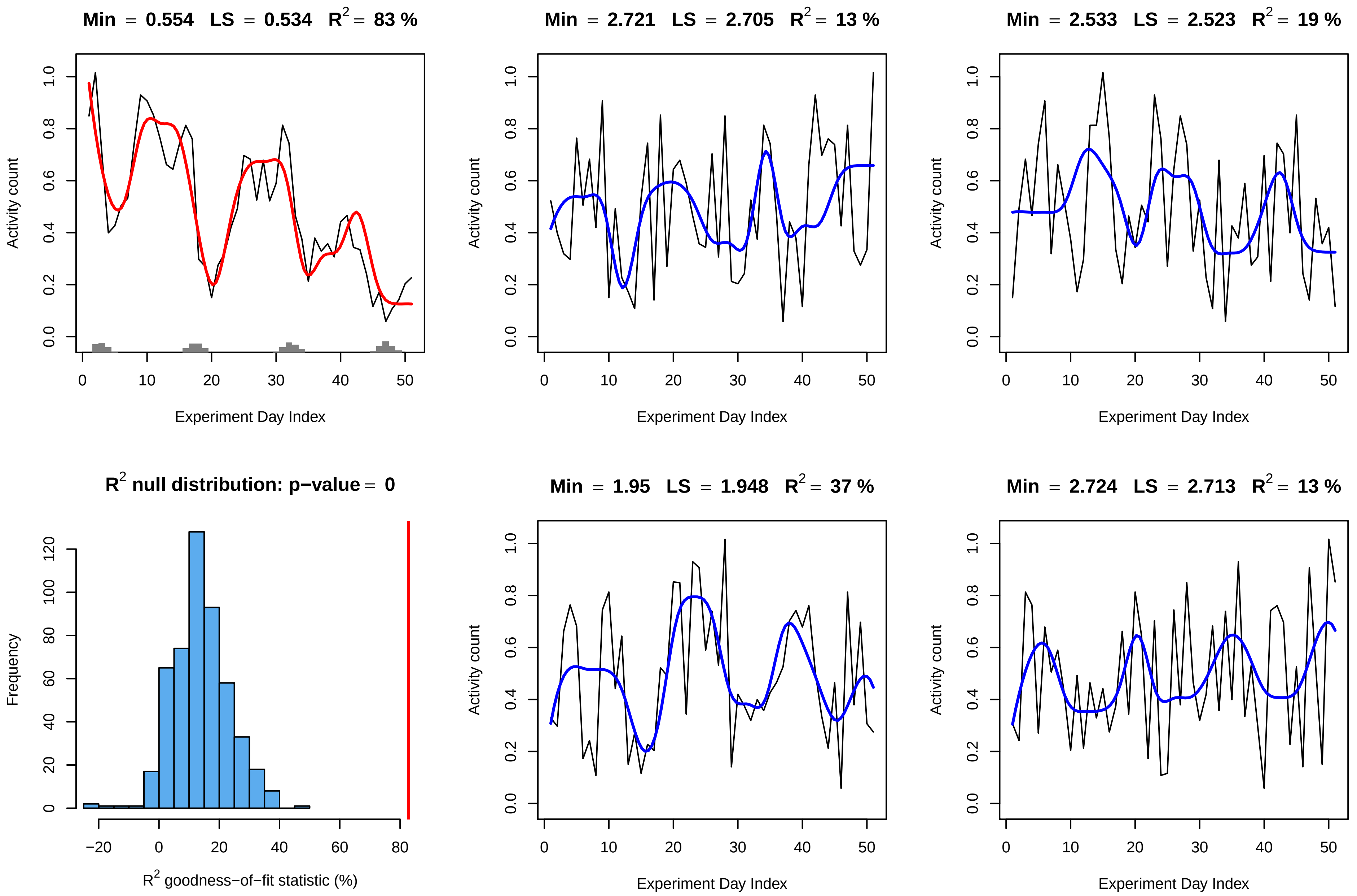

T24 : res.scheme2.exptdays1.51

Scheme 3: Day order preserved for each animal but with random start−day, selected for each animal separately for each simulation

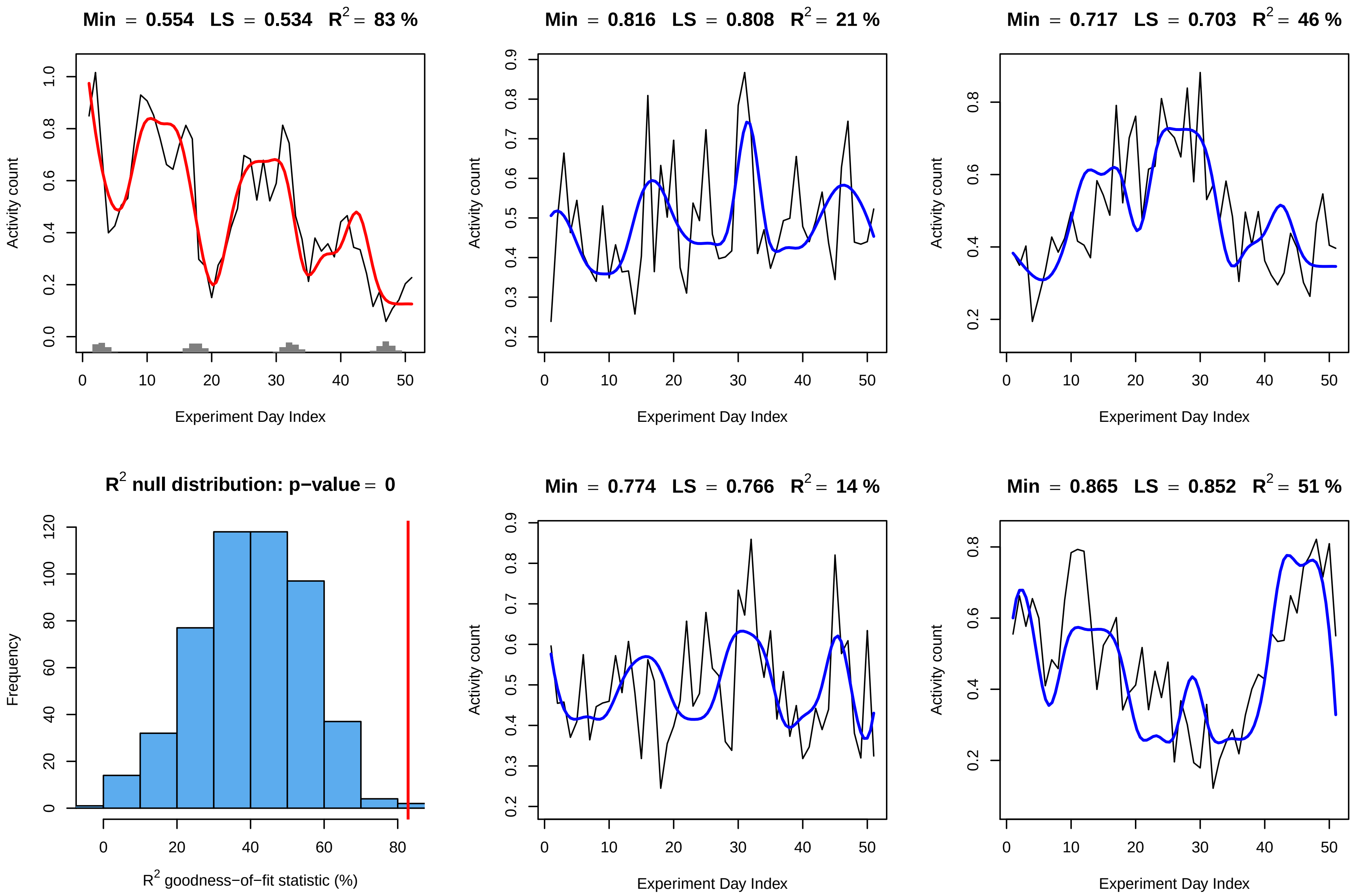

T24 : res.scheme3.exptdays1.51

Scheme 1: Days randomised for each animal separately for each simulation

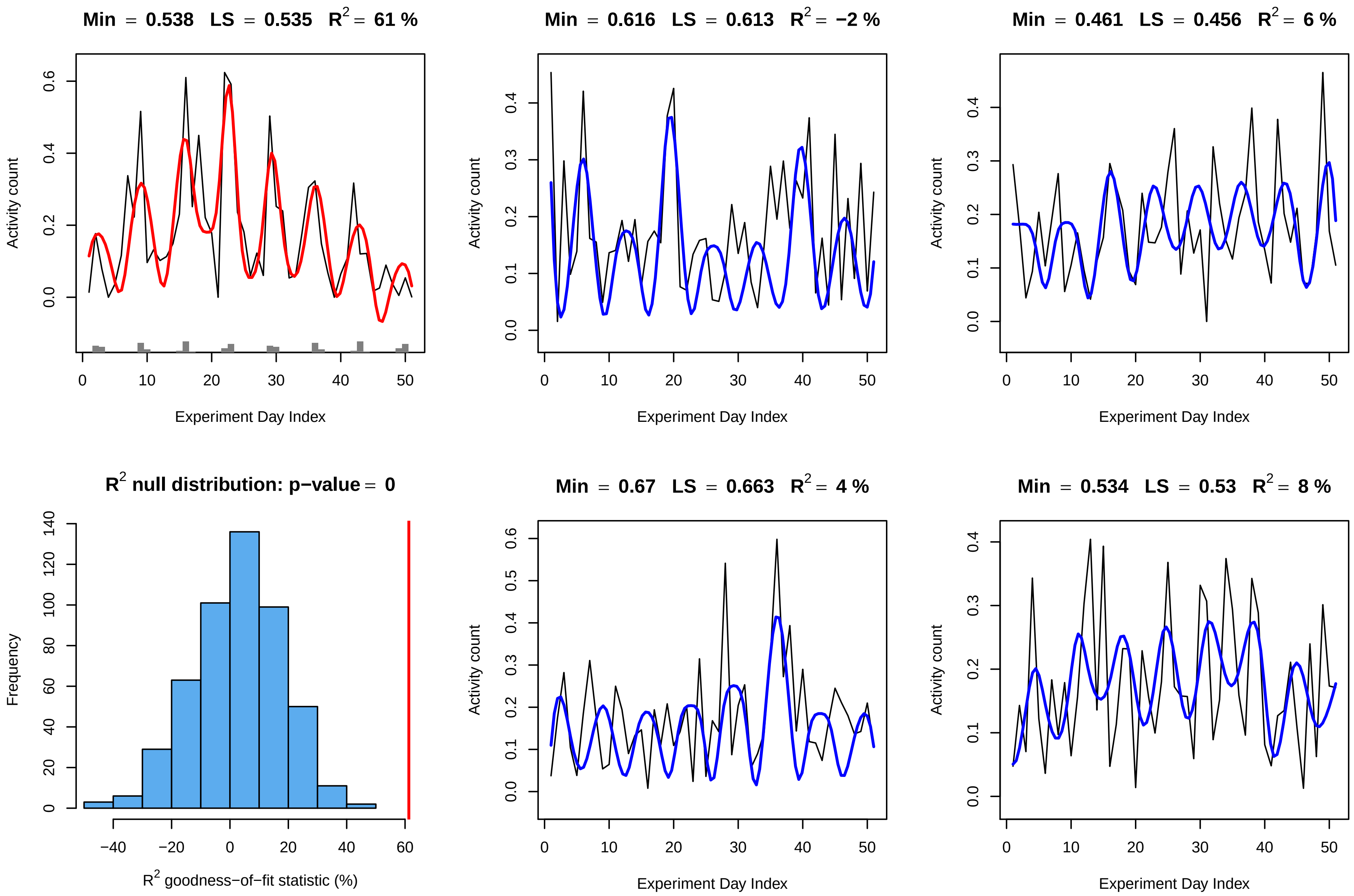

T23 : res.scheme1.exptdays1.51

Scheme 2: Days randomised for all animals collectively for each simulation

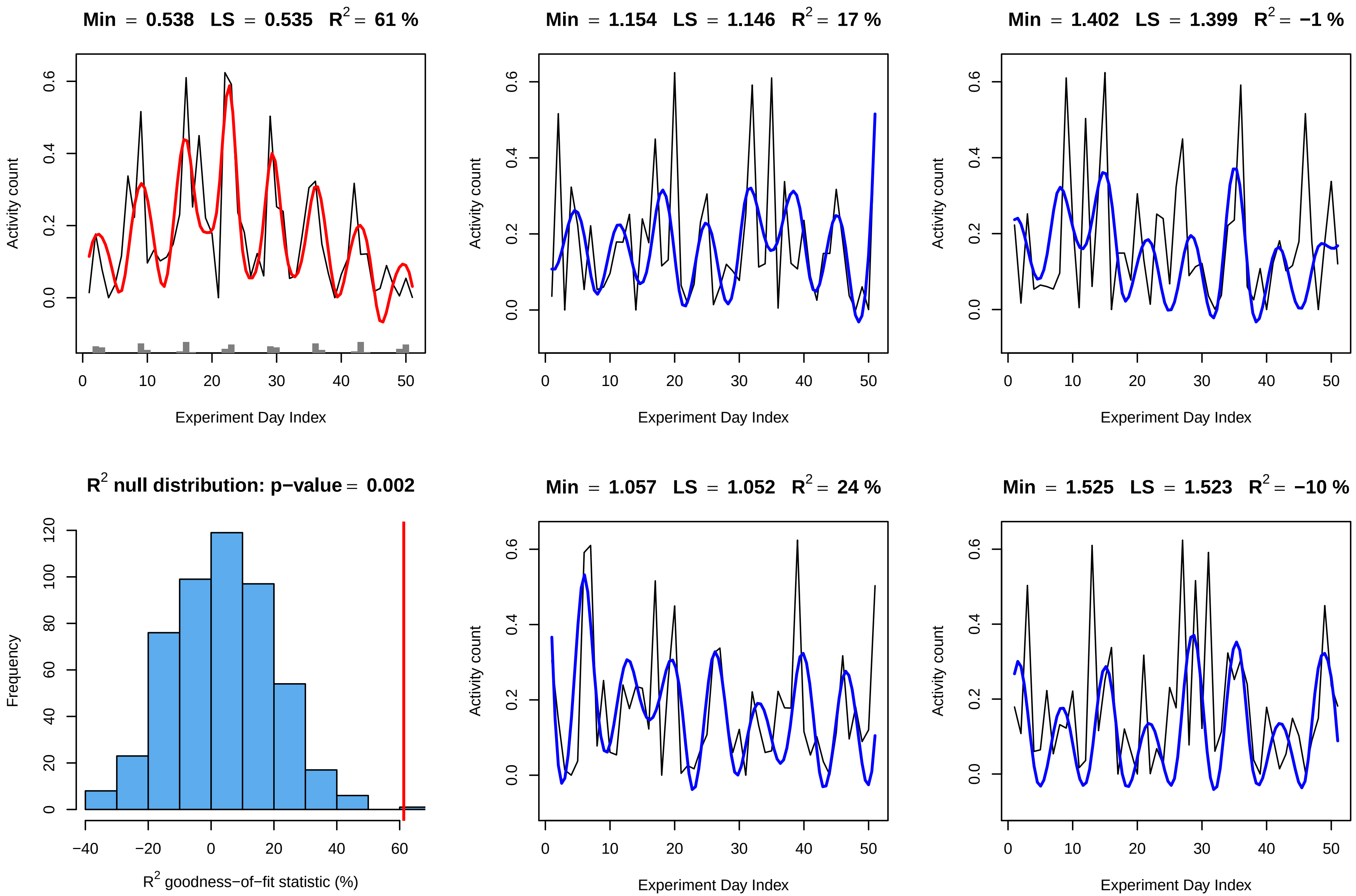

T23 : res.scheme2.exptdays1.51

Scheme 3: Day order preserved for each animal but with random start−day, selected for each animal separately for each simulation

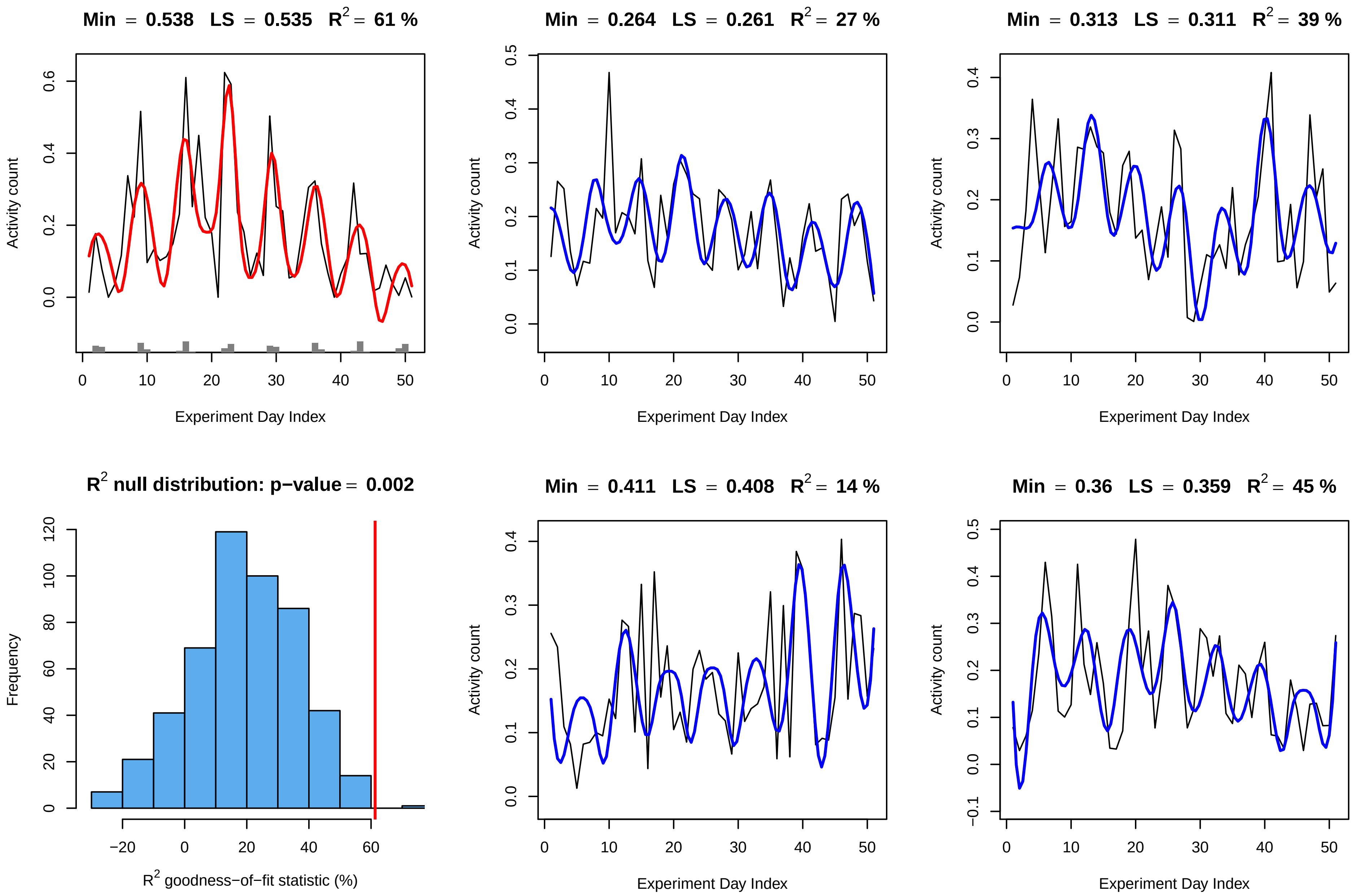

T23 : res.scheme3.exptdays1.51

Scheme 1: Days randomised for each animal separately for each simulation

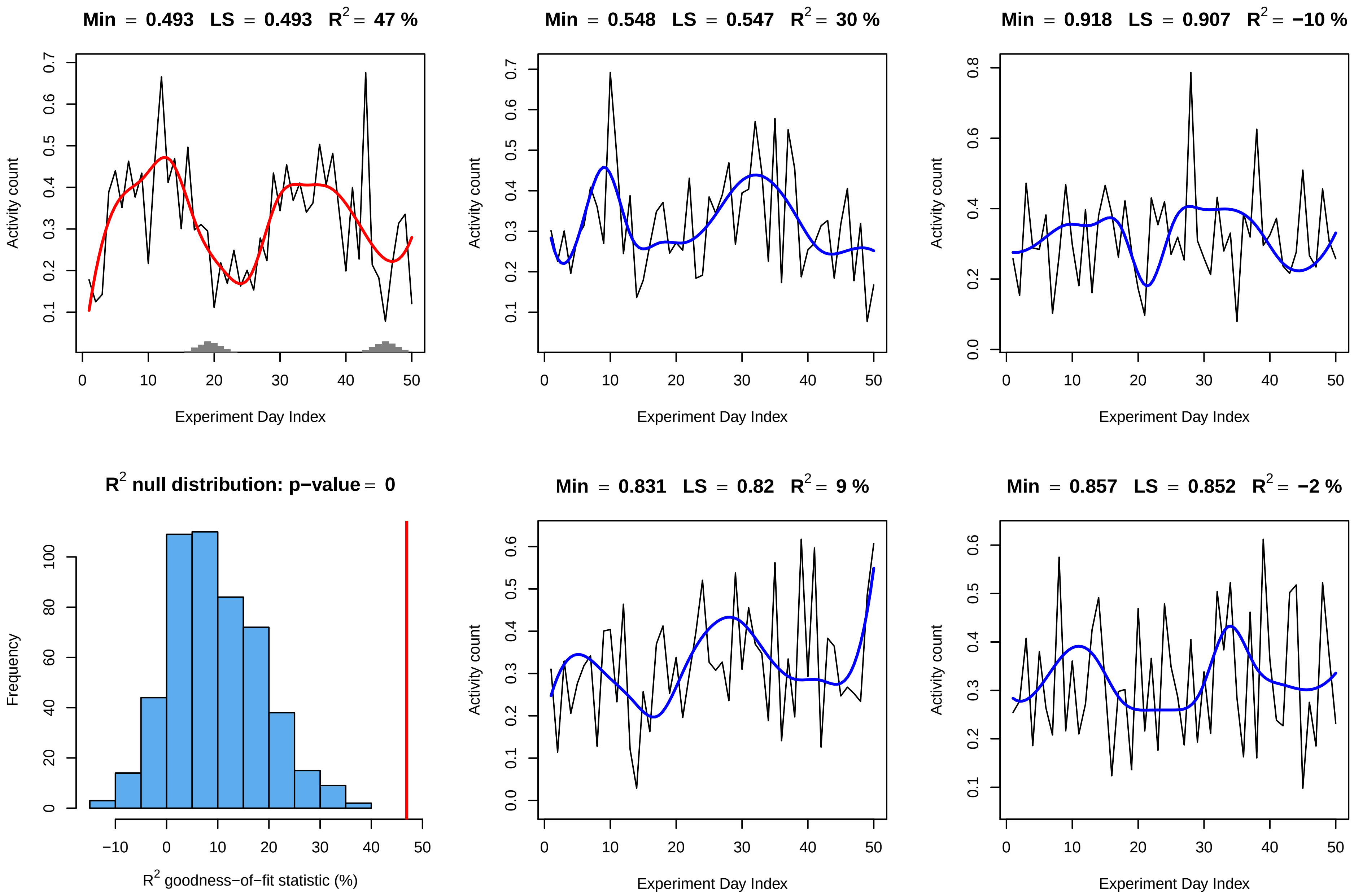

T24−3 : res.scheme1.exptdays2.51

Scheme 2: Days randomised for all animals collectively for each simulation

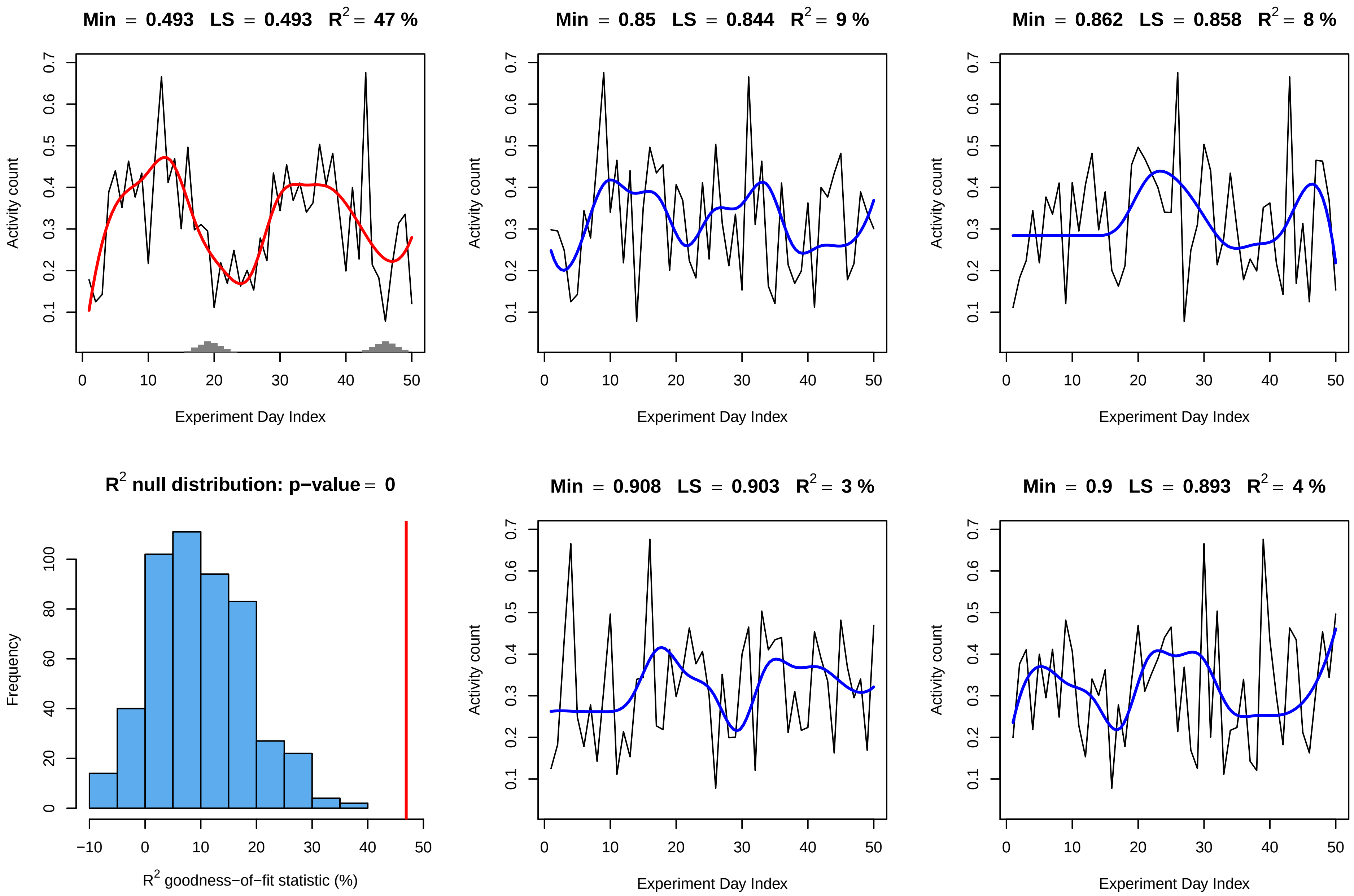

T24−3 : res.scheme2.exptdays2.51

Scheme 3: Day order preserved for each animal but with random start−day, selected for each animal separately for each simulation

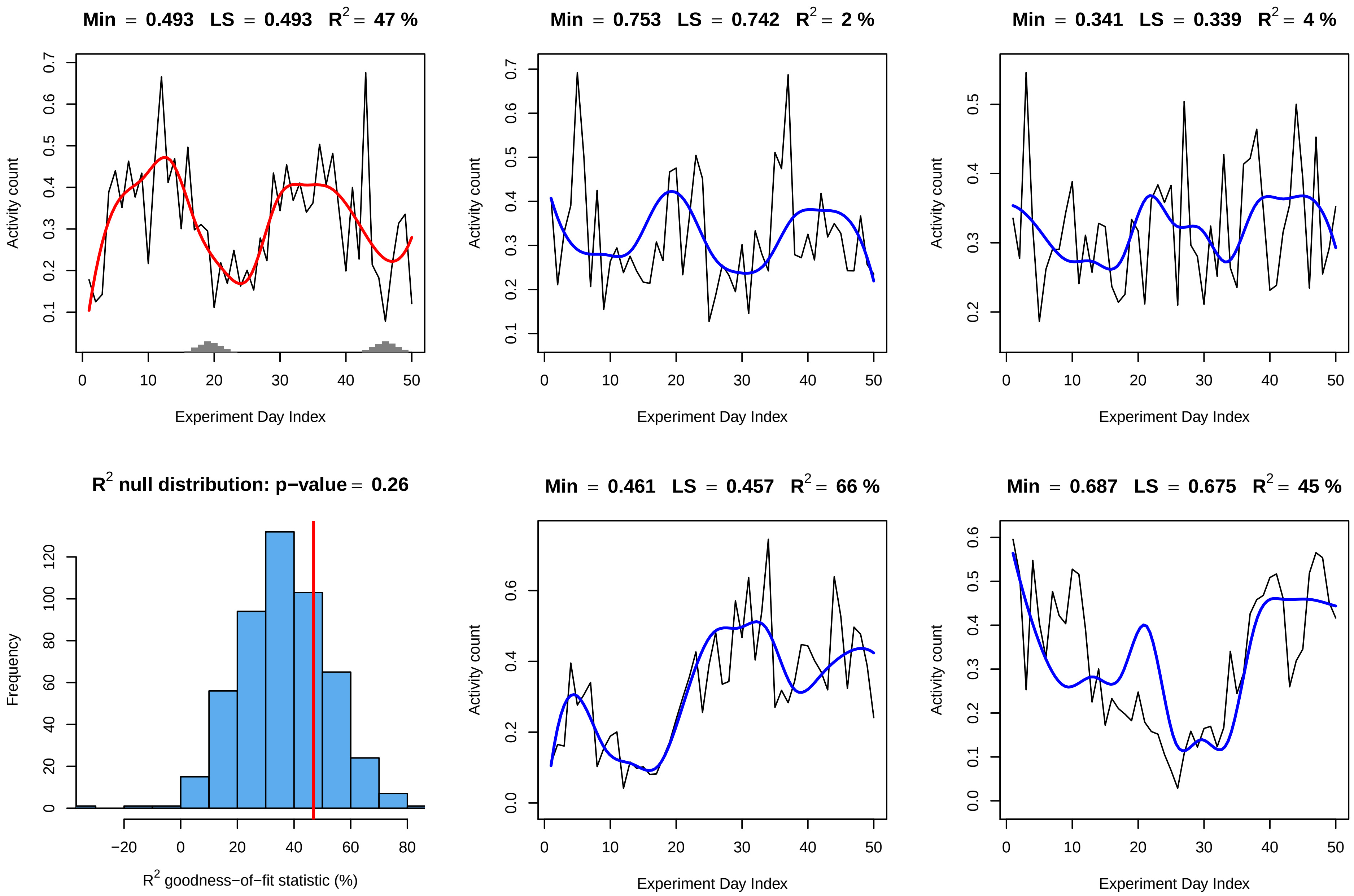

T24−3 : res.scheme3.exptdays2.51

Scheme 1: Days randomised for each animal separately for each simulation

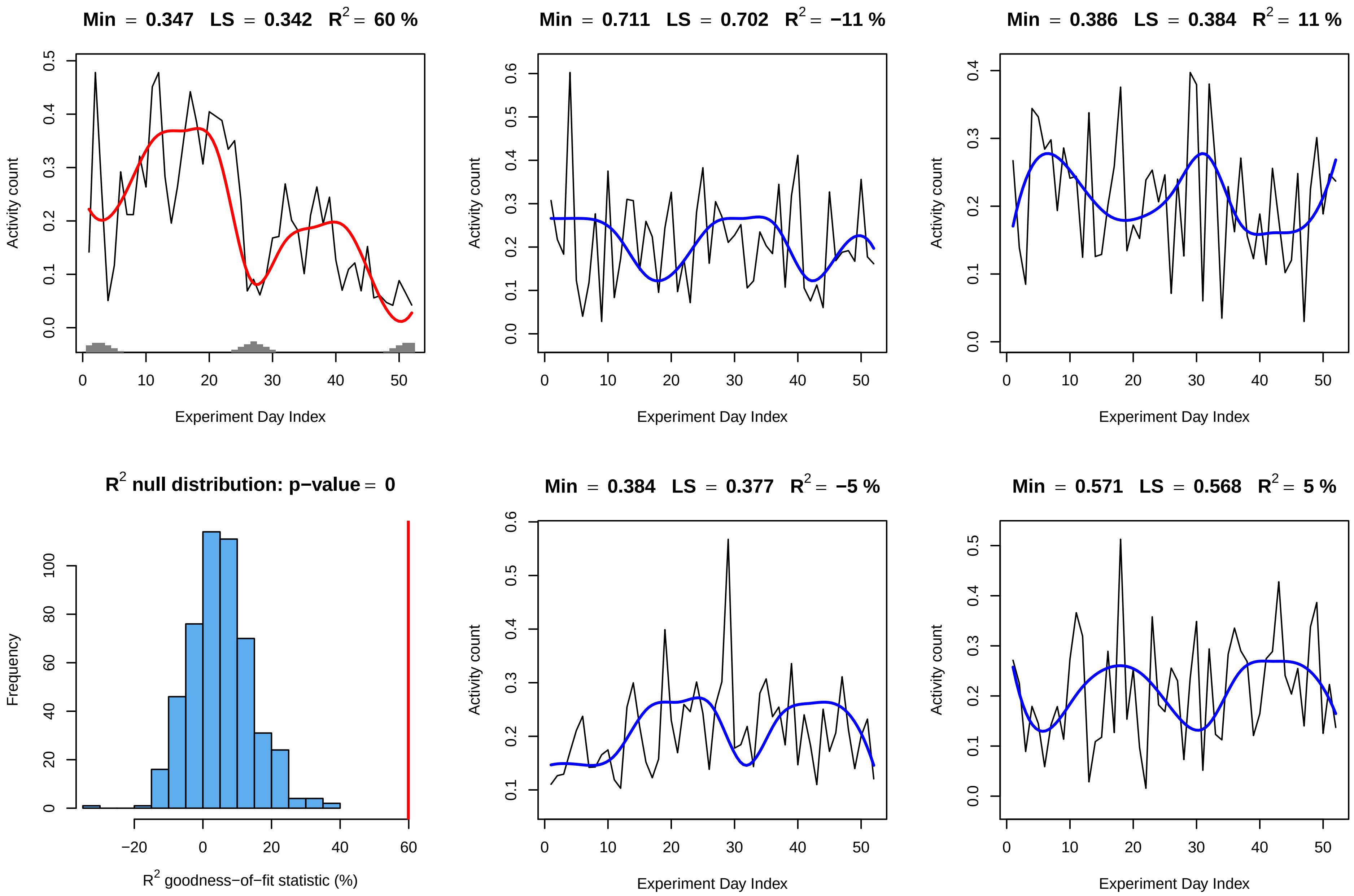

TIDAL_T12−25 : res.scheme1.exptdays1.52

Scheme 2: Days randomised for all animals collectively for each simulation

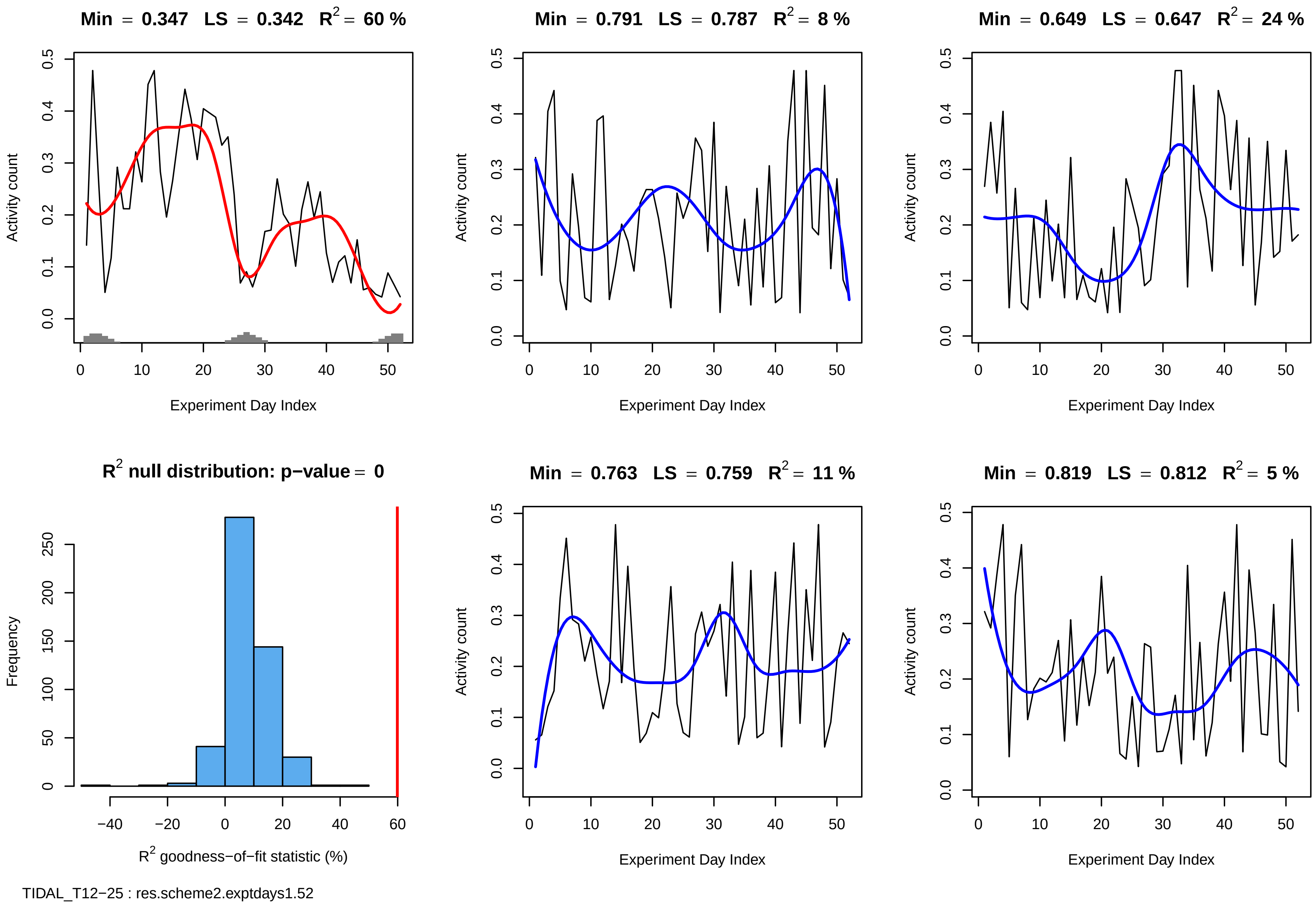

Scheme 3: Day order preserved for each animal but with random start−day, selected for each animal separately for each simulation

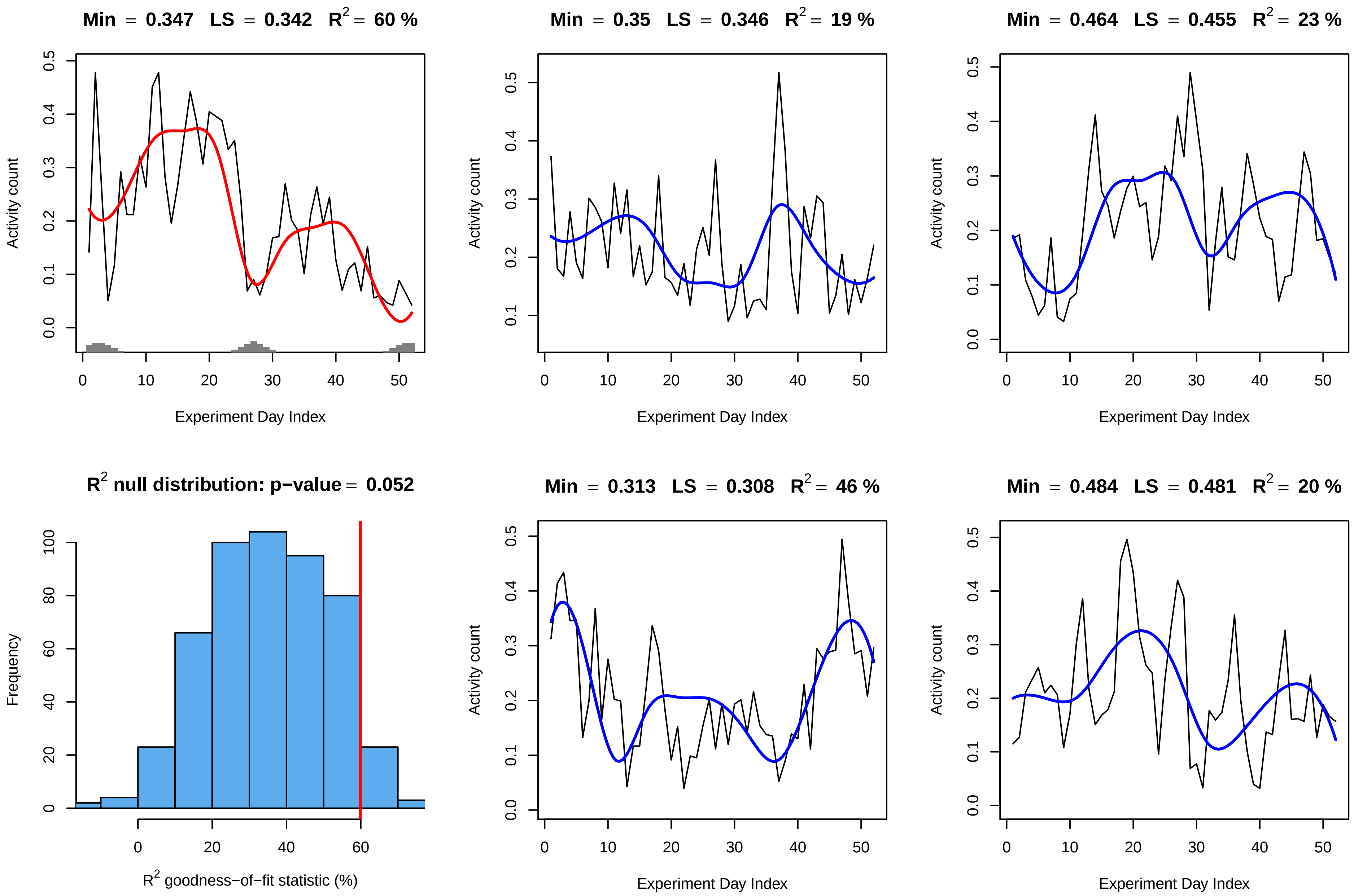

TIDAL_T12−25 : res.scheme3.exptdays1.52

Scheme 1: Days randomised for each animal separately for each simulation

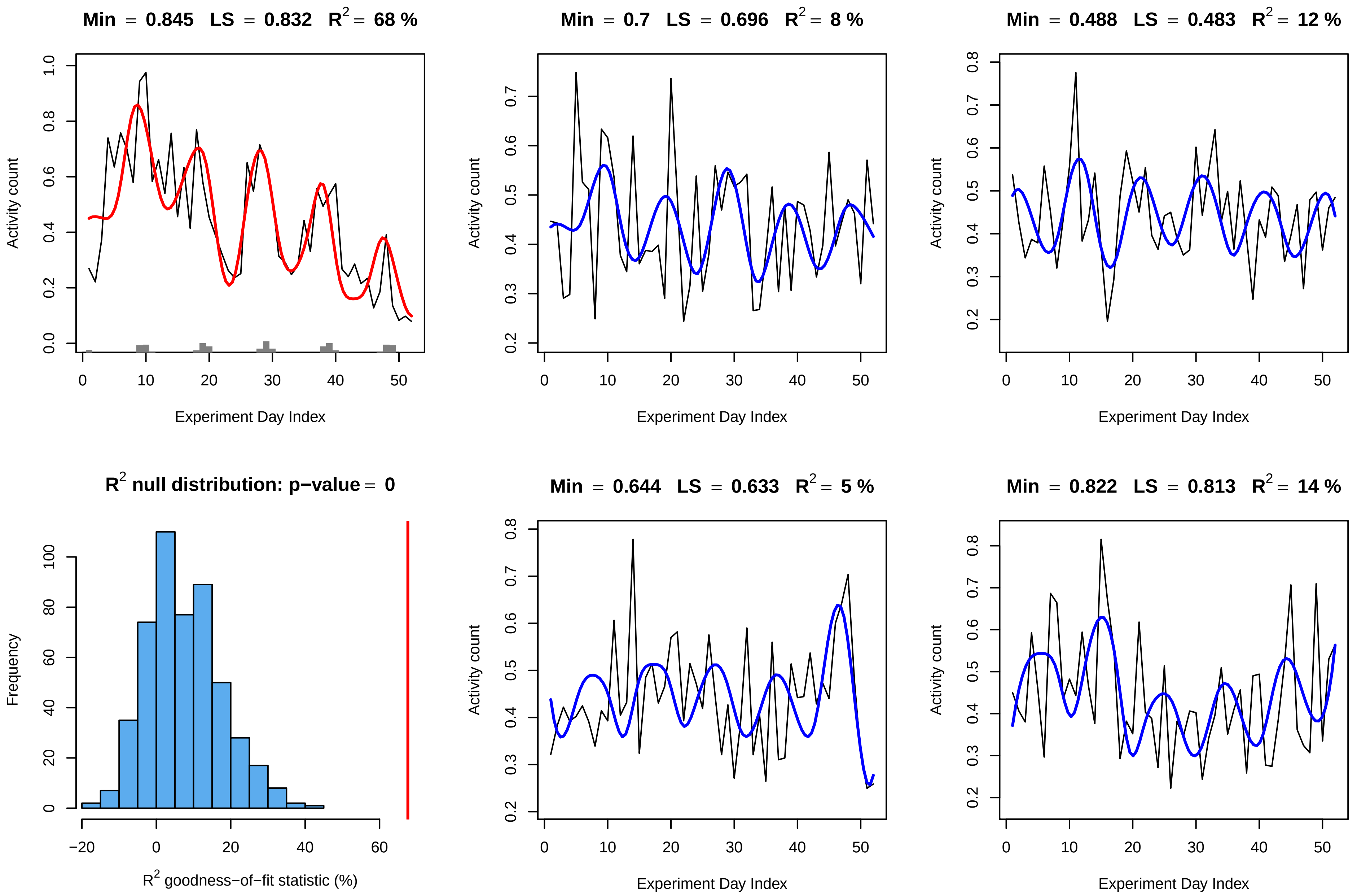

TIDAL_T12−65 : res.scheme1.exptdays2.53

Scheme 2: Days randomised for all animals collectively for each simulation

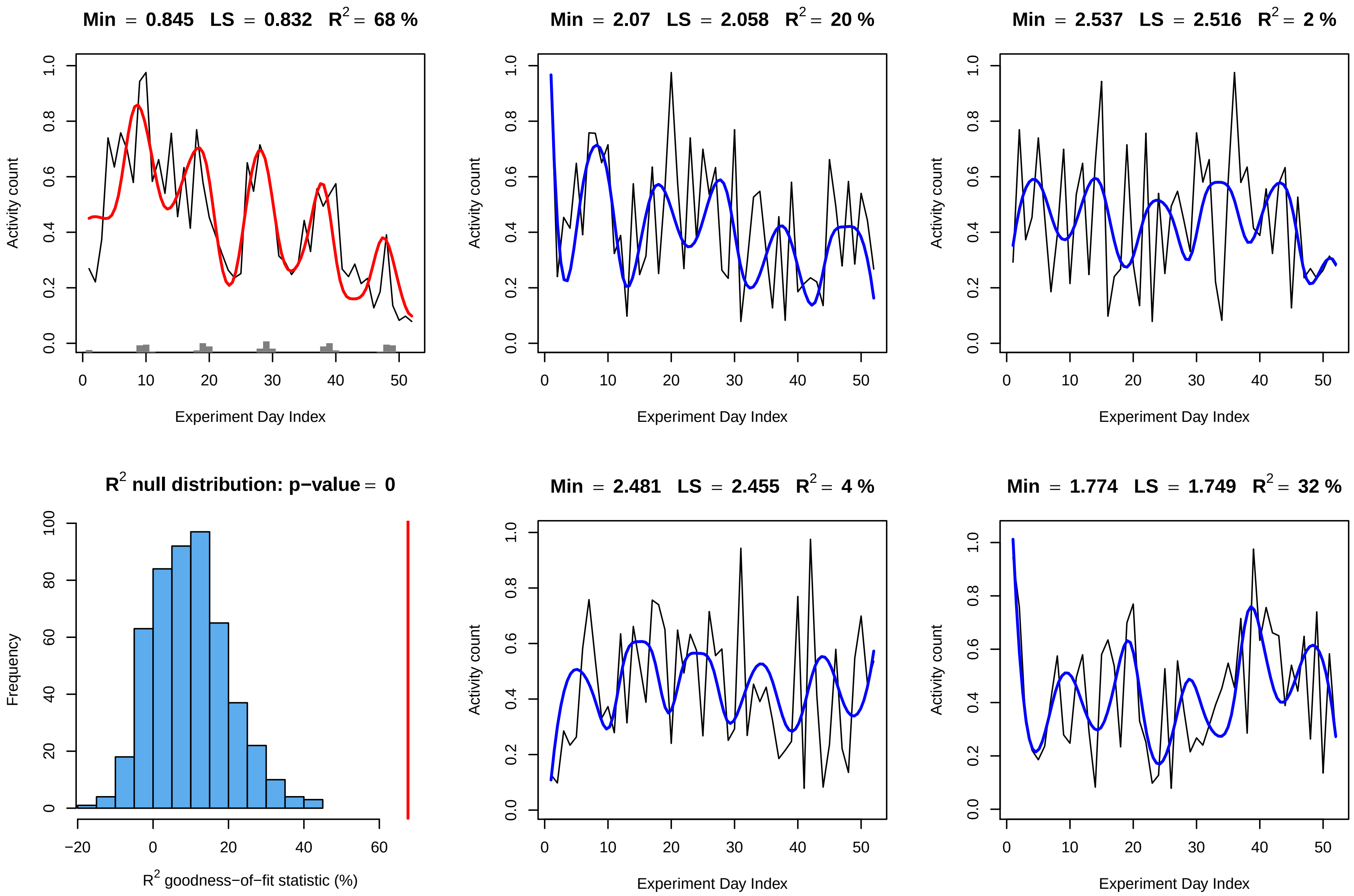

TIDAL_T12−65 : res.scheme2.exptdays2.53

Scheme 3: Day order preserved for each animal but with random start−day, selected for each animal separately for each simulation

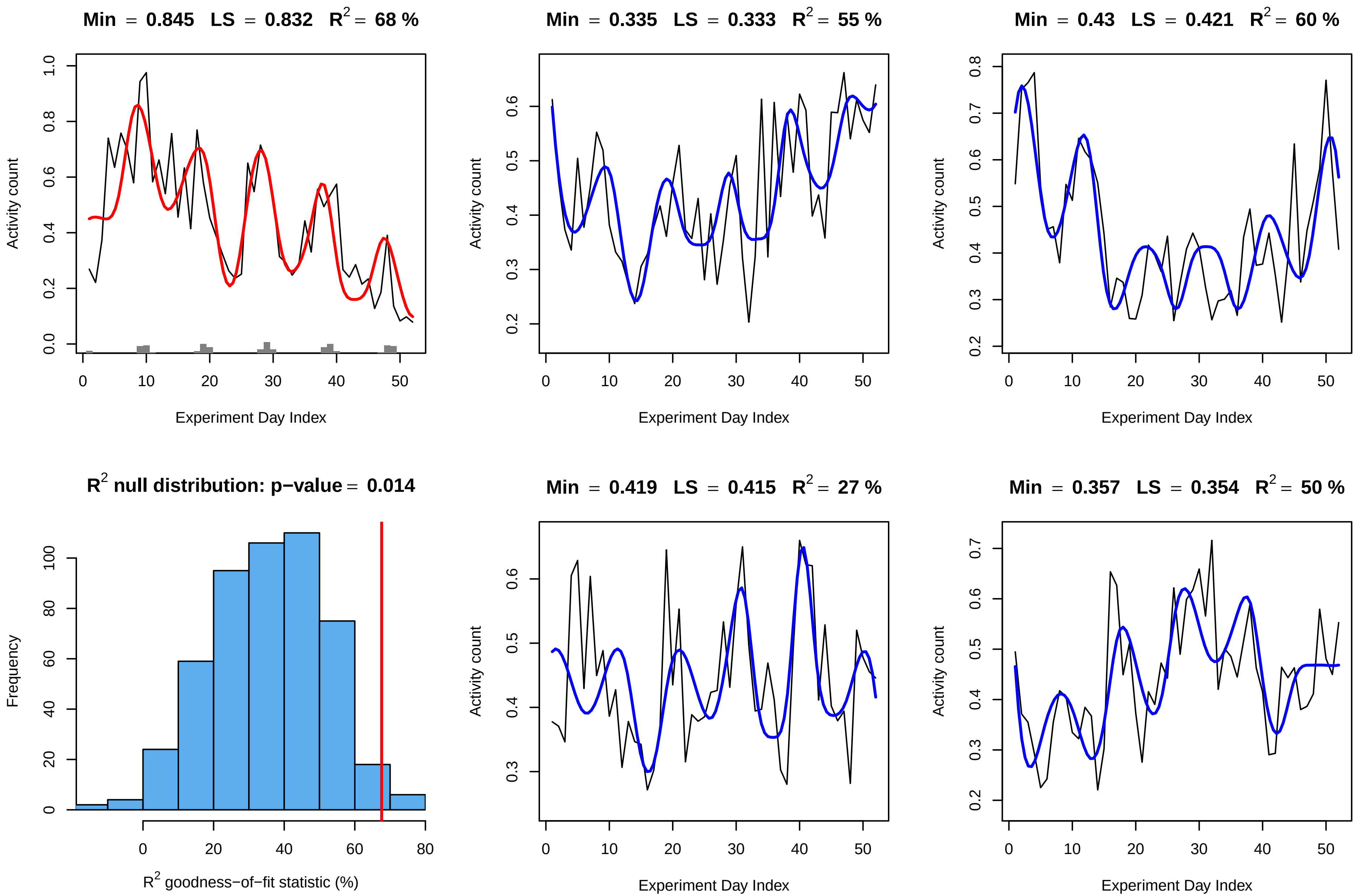

TIDAL_T12−65 : res.scheme3.exptdays2.53

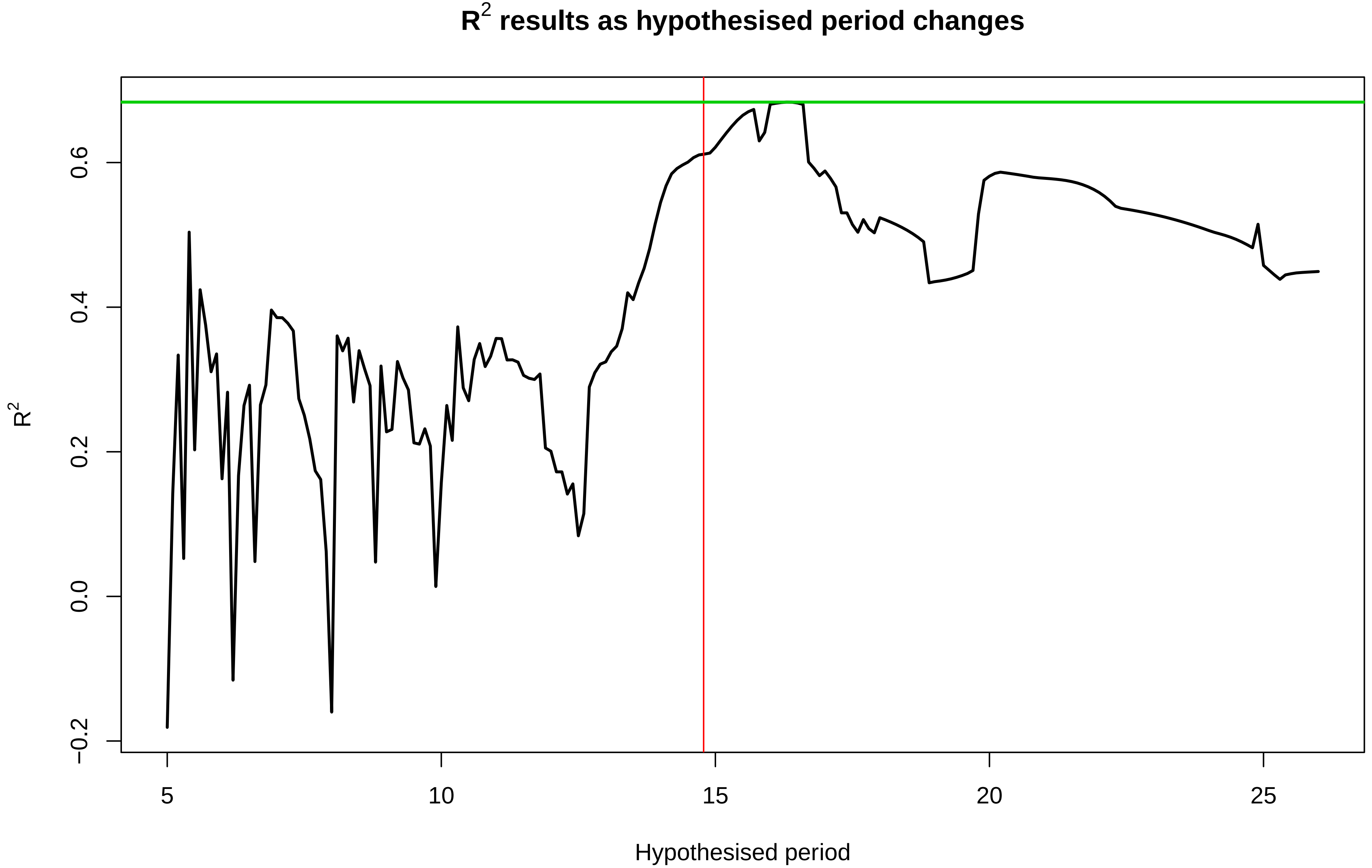

TDARK : periodres.fits.2.51

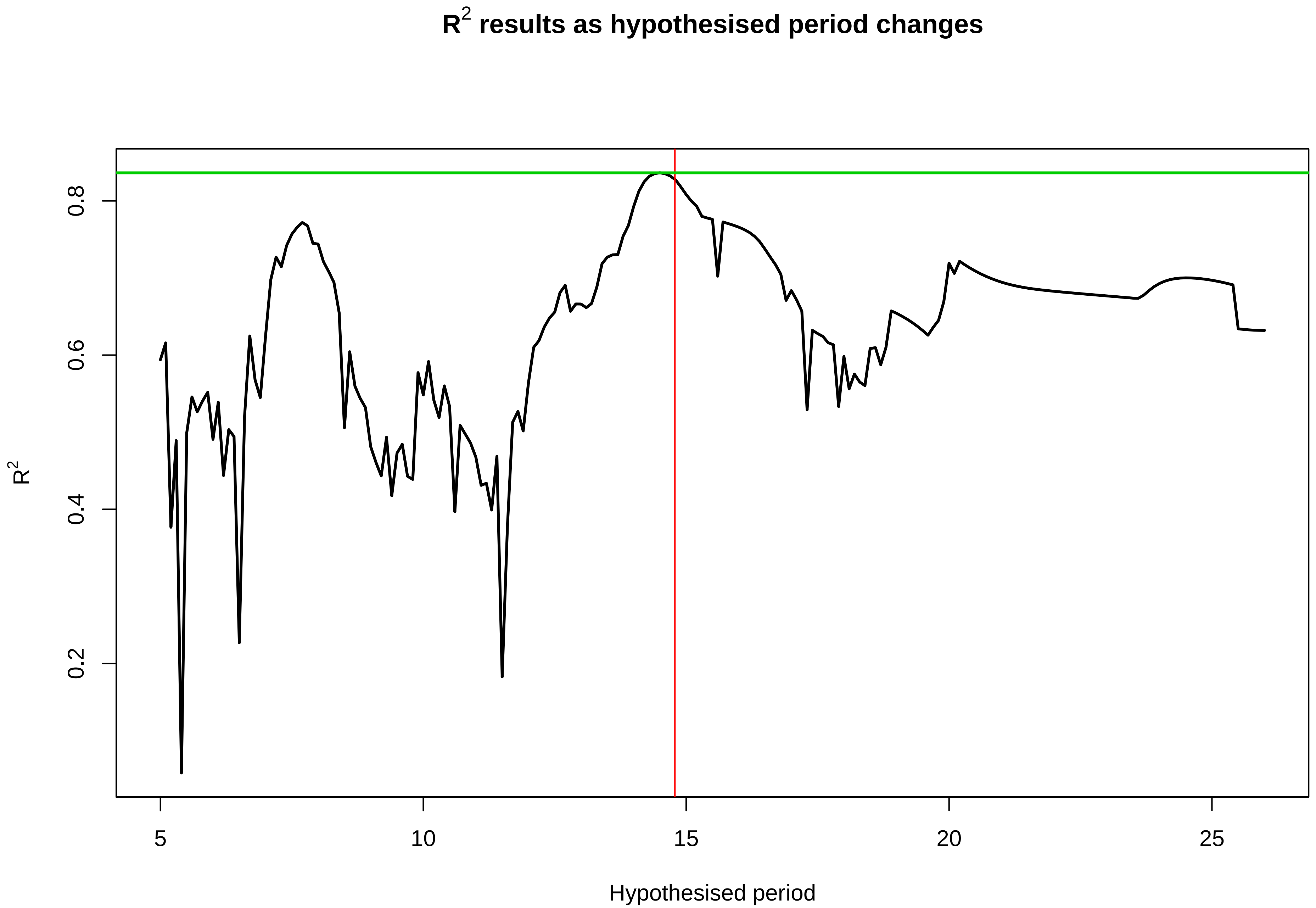

T24 : periodres.fits.1.51

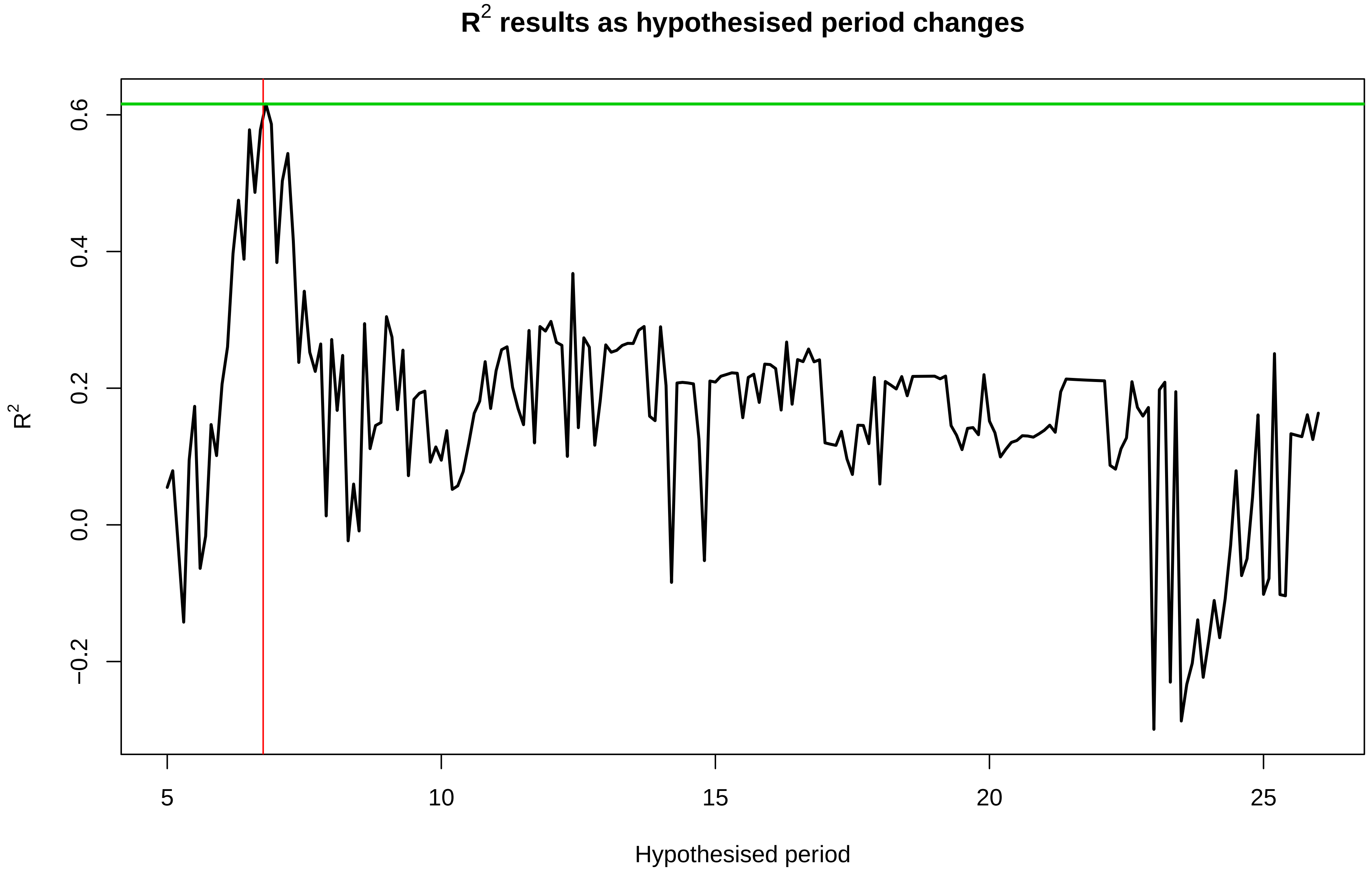

T23 : periodres.fits.1.51

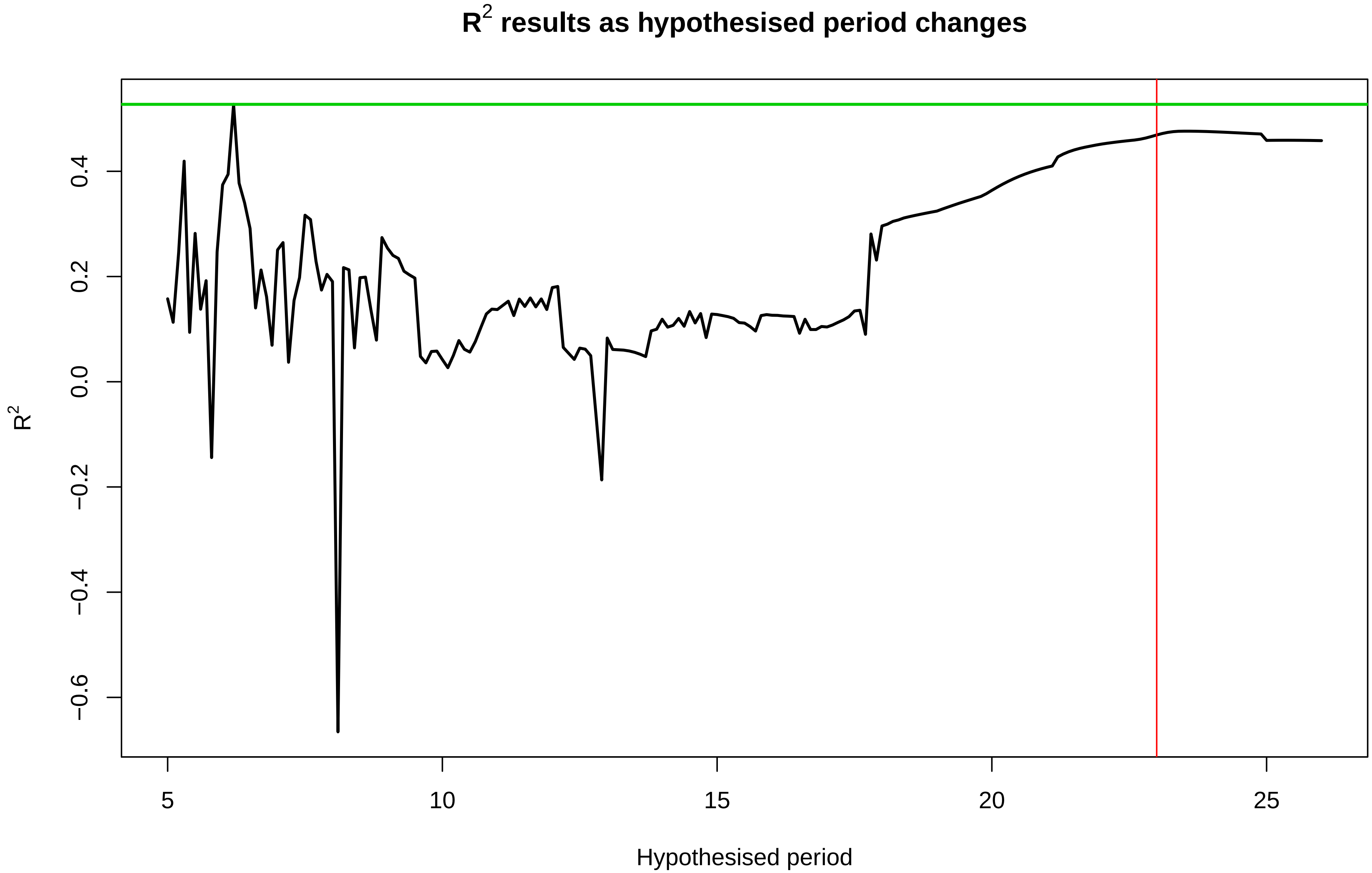

T24−3 : periodres.fits.2.51

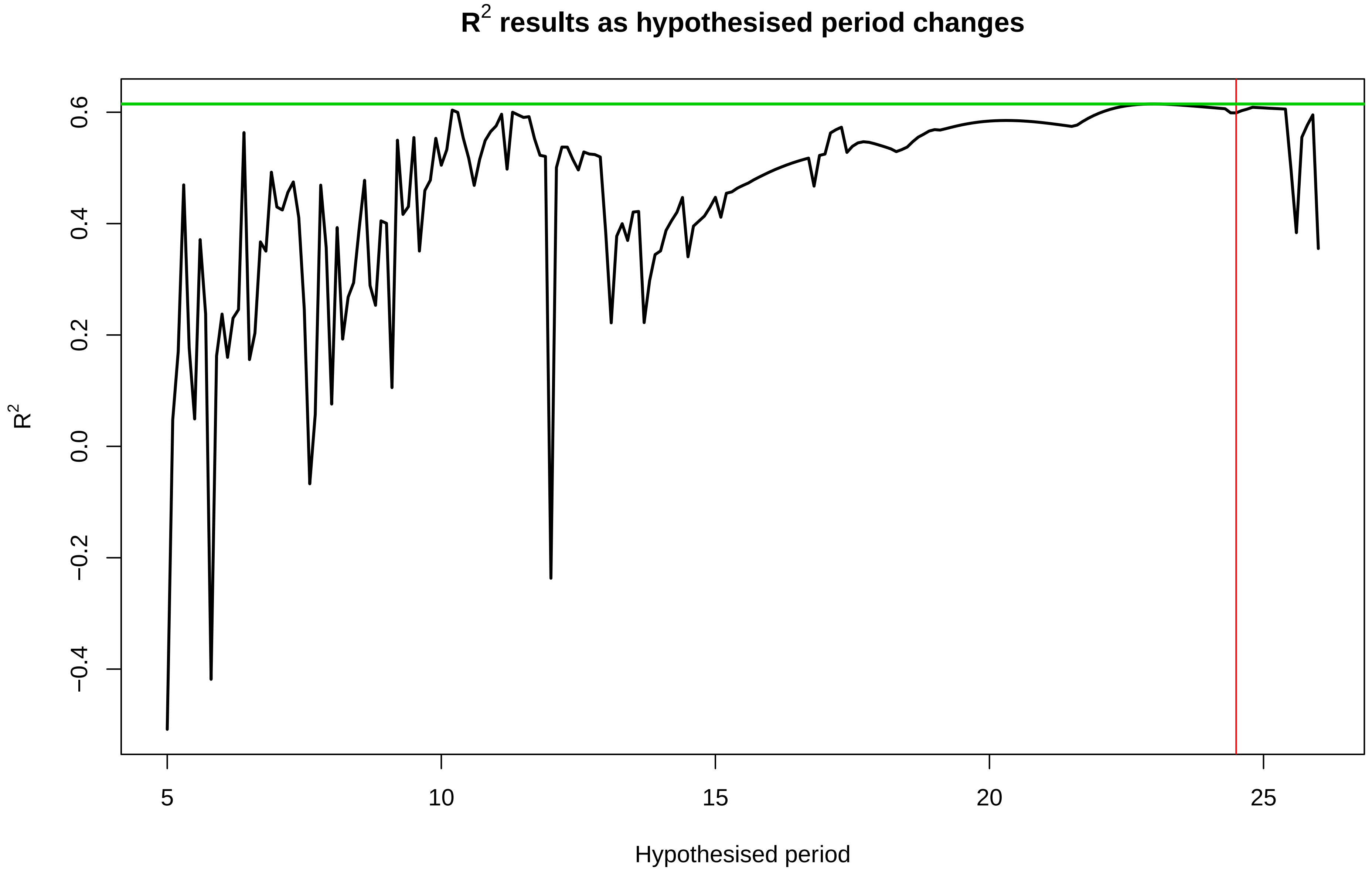

TIDAL_T12−25 : periodres.fits.1.52

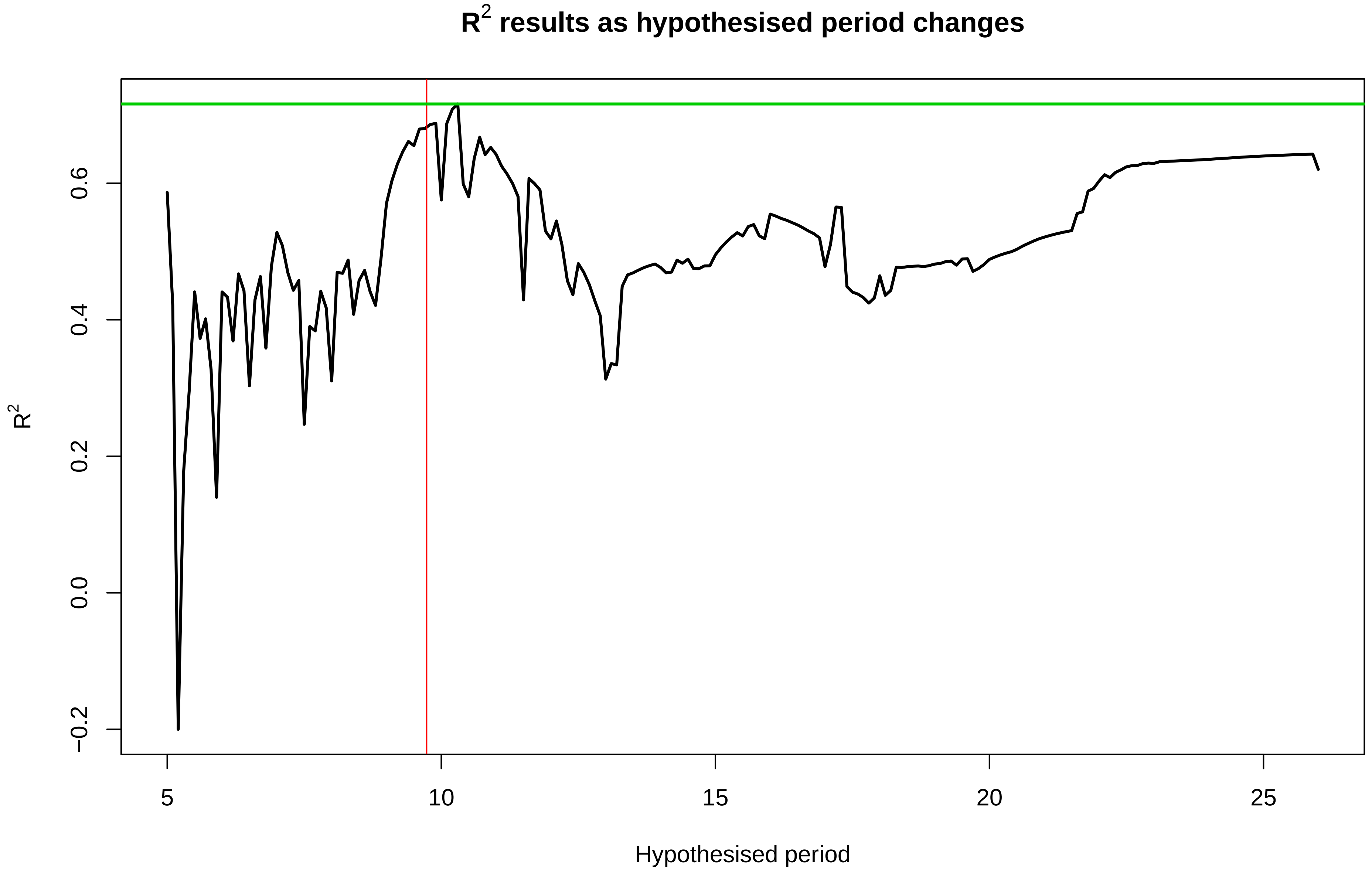

TIDAL_T12−65 : periodres.fits.2.53
